# NAT10-mediated ac4C modification of Lipin1 mRNA contributes to the pathogenesis of PWMI

**DOI:** 10.1172/jci.insight.193712

**Published:** 2025-08-08

**Authors:** Xinyu Li, Meng Zhang, Yanan Liu, Chunjie Guo, Yiwei Liu, Lei Han, Zhaowei Feng, Xiue Wei, Ruiqin Yao

**Affiliations:** 1 Department of Cell Biology and Neurobiology, Xuzhou Key Laboratory of Neurobiology, Xuzhou Medical University, Xuzhou, Jiangsu Province, China.; 2 Department of Neurology, and; 3 Department of Urology, Affiliated Hospital of Xuzhou Medical University, Xuzhou, Jiangsu Province, China.; 4 Department of Neurology, Second Affiliated Hospital of Xuzhou Medical University, Xuzhou, Jiangsu Province, China.

**Keywords:** Cell biology, Neuroscience, Behavior, Epigenetics, Neurodevelopment

## Abstract

Preterm white matter injury (PWMI) is a leading cause of cerebral palsy and chronic neurological disabilities in premature infants. It is characterized by defects in oligodendrocyte precursor cell (OPC) differentiation and dysmyelination. Currently, there are no effective therapeutic strategies available in clinical practice. Lipid homeostasis plays a crucial role in myelin development, yet the function of Lipin1 — a key phosphatidic acid phosphatase involved in phospholipid synthesis — remains unclear. In this study, we identified a significant downregulation of Lipin1 in OPCs from PWMI mice, which impaired OPC differentiation and myelin formation. Conversely, Lipin1 overexpression in these mice promoted OPC maturation and enhanced myelin development. We found evidence that *N*-acetyltransferase 10 (NAT10) acts as a regulator of Lipin1 expression through RNA pull-down and mass spectrometry. NAT10-mediated *N*^4^-acetylcytidine (ac4C) modification enhanced Lipin1 mRNA stability and translation, and NAT10 knockdown in OPCs impaired myelination, highlighting its crucial role in Lipin1-mediated myelination. Our study revealed that the downregulation of Lipin1 impaired OPC differentiation and myelination in PWMI, with NAT10-mediated ac4C modification playing a critical role in regulating Lipin1 expression. These findings highlight Lipin1 and NAT10 as promising therapeutic targets for treating myelination defects in PWMI, warranting further investigation into their potential in preterm birth–related neurological disorders.

## Abstract 2

NAT10-mediated ac4C modification regulates Lipin1 expression, contributing to the pathogenesis of PWMI and offering potential therapeutic targets for preterm birth-related neurological disorders.

## Graphical abstract

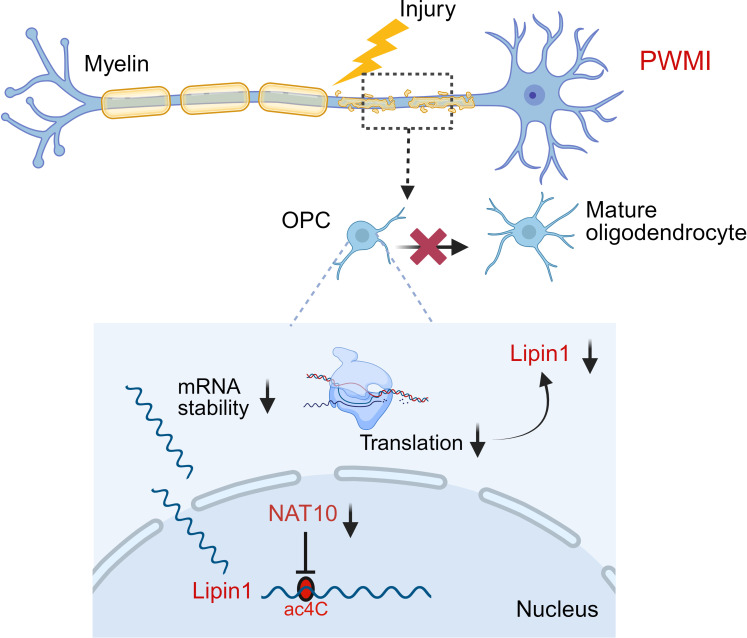

## Introduction

Preterm white matter injury (PWMI) is a leading cause of lifelong neurological impairments in preterm infants, including motor disabilities and cognitive deficits, with severe cases resulting in cerebral palsy (CP) (1–3). PWMI is primarily associated with perinatal hypoxia-ischemia (HI) and its severity increases with decreasing gestational age, which causes damage to the developing brain (4, 5). During HI conditions, oligodendrocyte progenitor cells (OPCs), which are essential for myelination, are particularly vulnerable, with limited antioxidant defenses and high mitochondrial oxygen consumption being the main factors contributing to their susceptibility (6–8). Although OPCs may undergo compensatory proliferation, their failure to differentiate leads to impaired axonal myelination, particularly in the corpus callosum (9–11). Despite advances in neonatal care, effective treatments for PWMI remain limited, making it crucial to investigate the key factors hindering OPC differentiation in PWMI for the prevention and treatment of this condition.

Emerging evidence suggests that lipid metabolism plays a vital role not only in the structural assembly of myelin membranes but also in regulating the lineage progression of OPCs (12–14). Among lipid metabolic enzymes, Lipin1, a phosphatidic acid phosphatase (PAP), catalyzes the conversion of phosphatidic acid (PA) to diacylglycerol (DG), an essential precursor for lipid biosynthesis and a second messenger in signaling cascades (15, 16). DG activates protein kinase C (PKC), which promotes OPC maturation and myelin production (17). In peripheral nervous system models, Lipin1 deficiency has been associated with demyelination due to PA accumulation (18). However, the role of Lipin1 in central nervous system myelination, particularly in the context of PWMI, remains poorly understood.

To address this gap, we investigated whether Lipin1 downregulation occurs in the brains of PWMI mice and assessed its functional consequences. We observed that Lipin1 expression is significantly downregulated following HI injury. Gain- and loss-of-function studies revealed that Lipin1 is a crucial driver of OPC differentiation and myelination under both physiological and HI conditions. To further explore the upstream regulatory mechanism of Lipin1 expression, we performed RNA pull-down combined with mass spectrometry analysis, leading to the identification of *N*-acetyltransferase 10 (NAT10) as a potential regulator. NAT10 is the only known enzyme mediating the *N*^4^-acetylcytidine (ac4C) modification, a rare RNA acetylation mark that enhances mRNA stability and translation in eukaryotic cells (19). Recent studies have shown that ac4C plays a crucial role in regulating gene expression during development and stress responses (20, 21). In our study, we demonstrated that NAT10-mediated ac4C modification enhances Lipin1 mRNA stability and translation. NAT10 knockdown significantly impaired OPC differentiation and myelination, highlighting what we believe to be a previously unrecognized epitranscriptomic mechanism in the pathogenesis of PWMI. Taken together, our study revealed a regulatory axis involving NAT10, ac4C, and Lipin1 that is critical for OPC maturation and myelin formation. These findings not only expand our understanding of the molecular basis of PWMI but also point to potential therapeutic targets for the prevention and treatment of PWMI.

## Results

### Myelination deficits and behavioral abnormalities in PWMI mice.

**Figure 1:**
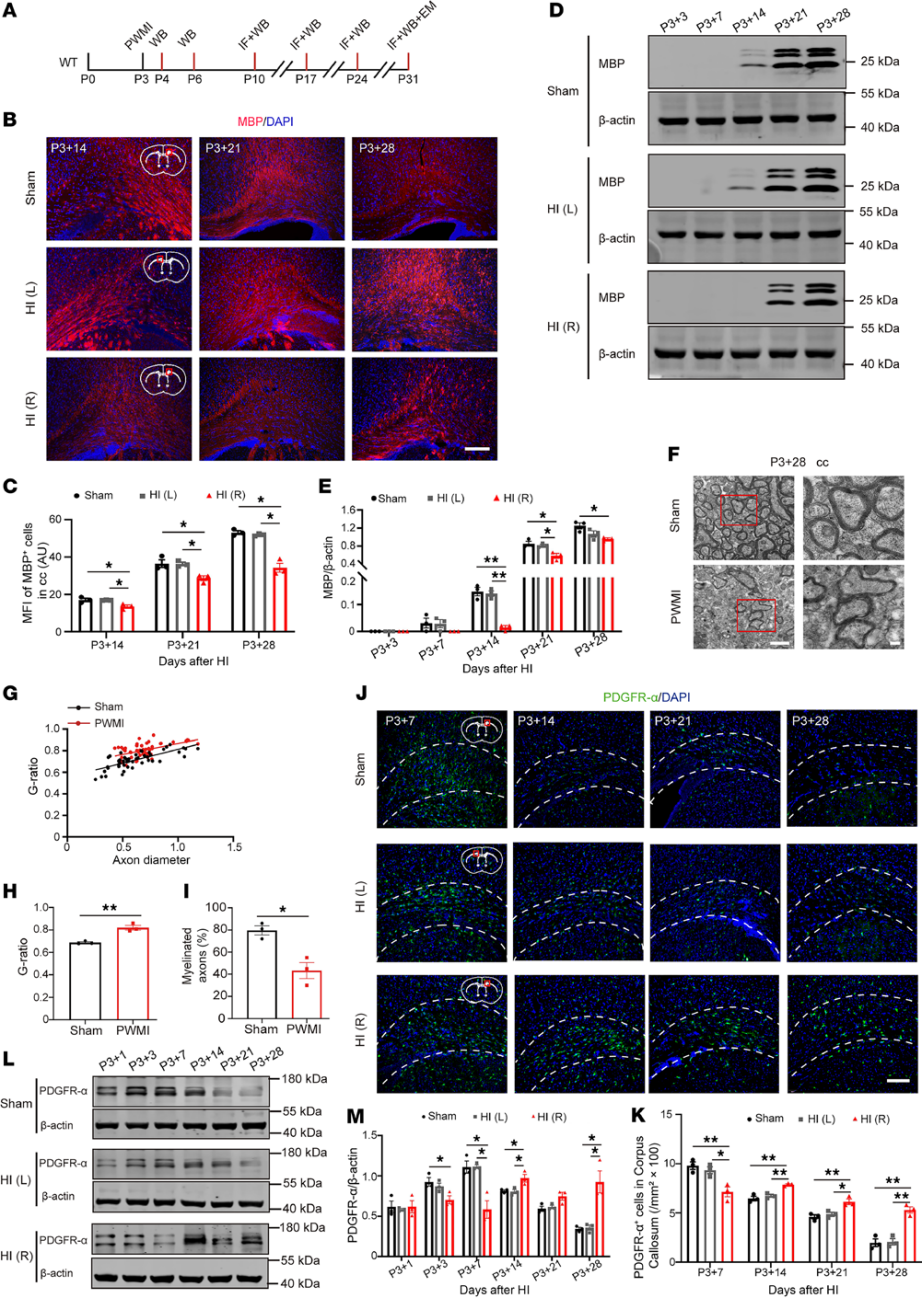
Myelination deficits in PWMI model mice. (**A**) Experimental diagram. (**B** and **C**) The MBP levels in corpus callosum were detected by immunofluorescent staining at 14, 21, and 28 days after modeling and statistical analysis. (**D** and **E**) The changes in MBP content in the corpus callosum region of mice at 3, 7, 14, 21, and 28 days after modeling were detected by Western blot analysis and statistical analysis. Representative of 3 independent experiments. (**F**) TEM images of the myelin sheath of the corpus callosum (cc) of 2 groups of mice. The high-magnification images are zoomed-in views of the red boxes in the low-magnification images. Scale bars: 1 μm (left) and 200 nm (right). (**G** and **H**) G-ratio distribution chart and statistical chart. (**I**) Percentage diagram of the number of myelinated axons. The number of evaluated myelinated axons was *n* = 50/mouse. (**J** and **K**) The level of PDGFR-α and the number of PDGFR-α^+^ cells in the corpus callosum of PWMI mice at different time points were detected by immunofluorescent staining. (**L** and **M**) Western blot analysis of PDGFR-α levels in corpus callosum of PWMI mice at different time points and statistical analysis. Representative of 3 independent experiments. Statistics: 1-way ANOVA with LSD post hoc analysis (**C**, **E**, and **K**), 2-tailed Student’s *t* test (**H** and **I**), and 1-way ANOVA with Dunnett’s test (**M**). HI (R), ischemic side; HI (L), ischemic side. Scale bar: 100 μm. *n* = 3. The results are expressed as mean ± SEM.

To investigate myelination after HI injury, we assessed myelin development at multiple time points using immunofluorescence (IF), Western blotting (WB), and transmission electron microscopy (TEM) (Figure 1A). IF analysis showed a significant decrease in myelin basic protein (MBP) fluorescence intensity in the ischemic corpus callosum [HI (R)] of PWMI mice compared with the contralateral side [HI (L)] and the Sham group (Figure 1, B and C). Consistently, WB confirmed decreased MBP levels in HI (R) from 14 days after HI was induced (P3+14) to P3+28 (Figure 1, D and E). TEM analysis revealed thinner myelin sheaths, an increased G-ratio, and a reduced density of myelinated axons in PWMI mice compared with Sham mice (Figure 1, F–I). These results demonstrate that HI injury leads to significant myelination deficits in the corpus callosum. Platelet-derived growth factor receptor α (PDGFR-α) was used as a marker to monitor OPC differentiation. In the Sham group, PDGFR-α levels decreased over time as OPCs differentiated into mature oligodendrocytes. In contrast, PWMI mice exhibited a distinct pattern; PDGFR-α was significantly reduced at P3+7 but remained abnormally elevated at P3+14, P3+21, and P3+28 in HI (R) compared with both Sham and HI (L) sides (Figure 1, J and K). Moreover, WB analysis showed that PDGFR-α expression decreased during the acute phase (P3+1 to P3+7) but paradoxically increased during the subacute phase (P3+7 to P3+21) (Figure 1, L and M). These findings suggest that HI injury not only delays OPC differentiation but also leads to dysregulation of the OPC pool over time.

To evaluate the functional consequences of myelination deficits, behavioral tests were conducted, including the Morris water maze (MWM), Y-maze, open field, rotarod, and gait analysis. PWMI mice exhibited reduced body weight (Supplemental Figure 1A; supplemental material available online with this article; https://doi.org/10.1172/jci.insight.193712DS1). In the MWM, PWMI mice had a longer latency to find the platform (Supplemental Figure 1, B and C), crossed the original platform location fewer times, and spent less time in the target quadrant after platform removal compared with Sham mice (Supplemental Figure 1, D and E). In the Y-maze, they entered the new arm fewer times and spent less time there (Supplemental Figure 1, F and G). The open field test showed reduced total distance and average speed in PWMI mice (Supplemental Figure 1, H–J). In the rotarod, PWMI mice ran for less time compared with Sham (Supplemental Figure 1K). Gait analysis revealed longer stance, swing, and propulsion durations for both forepaws and hindpaws in PWMI mice (Supplemental Figure 1, L–N). These findings indicate deficits in spatial memory and motor function after early postnatal HI.

### Lipin1 expression is reduced in OPCs of PWMI mice.

**Figure 2:**
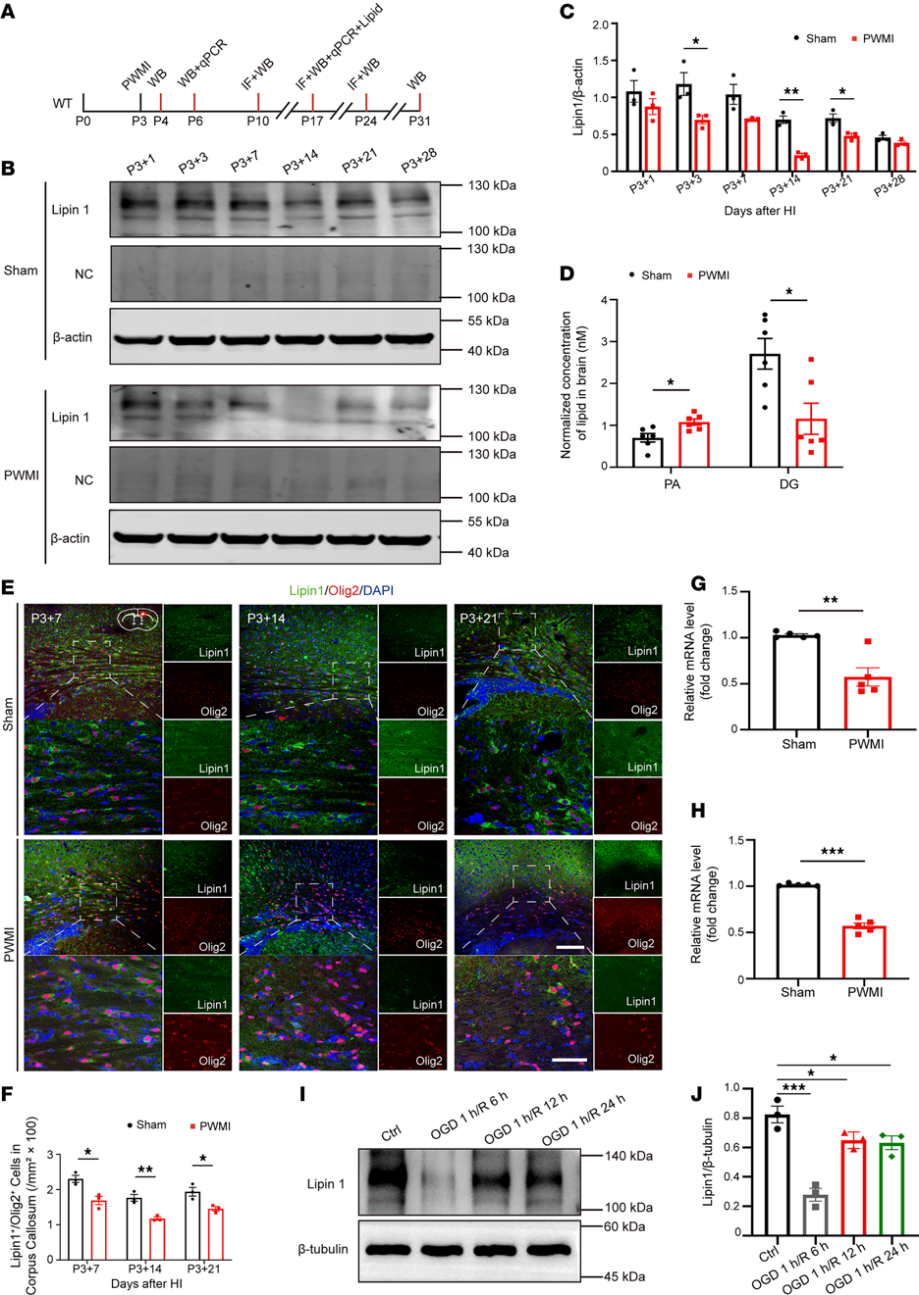
Reduced expression of Lipin1 in the OPCs of PWMI mice. (**A**) Experimental diagram. (**B** and **C**) Western blot analysis was conducted to detect changes in Lipin1 levels in the corpus callosum of mice from both groups at different time points. NC, negative control (sample incubated with secondary antibody only, no primary antibody), confirming the specificity of the Lipin1 signal. Statistical analysis was performed (*n* = 3). Representative of 3 independent experiments. (**D**) PA and DG levels in the brain tissue of mice from 2 groups (*n* = 6). (**E** and **F**) Lipin1 levels (green) in oligodendrocytes (Olig2, red) at different time points were detected by immunofluorescent staining, followed by statistical analysis (*n* = 3). Scale bars: 100 μm (low magnification) and 25 μm (high magnification). (**G** and **H**) mRNA levels of Lipin1 in the P3+3 and P3+14 corpus callosum of 2 groups of mice (*n* = 5). (**I** and **J**) Western blot analysis of Lipin1 levels in primary OPCs from control and OGD groups of mice, followed by statistical analysis (*n* = 3). Representative of 3 independent experiments. Statistics: 2-tailed Student’s *t* test (**C**, **D**, and **F**–**H**) and 1-way ANOVA with LSD post hoc analysis (**J**). The results are expressed as mean ± SEM. *P < 0.05; **P < 0.01; ***P < 0.001.

Lipin1, a PAP, is critical for maintaining lipid homeostasis, an essential process for myelination (22). WB analysis showed that in Sham mice, Lipin1 levels in the corpus callosum decreased from early postnatal stages, stabilizing by P3+14 (Figure 2, A–C). In contrast, PWMI mice exhibited significantly reduced Lipin1 expression at P3+3, P3+14, and P3+21 (Figure 2, B and C). As an internal control for antibody specificity, a negative control (secondary antibody only, no primary antibody) was included in the WB assay, which showed no visible bands, confirming the specificity of Lipin1 detection (Figure 2B). Additionally, PA levels were elevated and DG levels were decreased in PWMI mouse brains (Figure 2D), suggesting disrupted lipid metabolism. IF staining revealed a marked reduction in Lipin1^+^/Olig2^+^ double-positive cells in the corpus callosum of PWMI mice (Figure 2, E and F). Lipin1 expression at P3+3 and P3+14 was notably lower in PWMI mice (Figure 2, G and H). Consistently, in primary OPCs subjected to oxygen-glucose deprivation (OGD), Lipin1 levels were significantly reduced at multiple time points after reoxygenation (Figure 2, I and J). These results highlight that HI injury leads to a significant decrease in Lipin1 expression in the corpus callosum and OPCs, implicating its role in impaired myelination.

### Lipin1 regulates OPC differentiation, myelination, and behavioral changes in mice.

**Figure 3:**
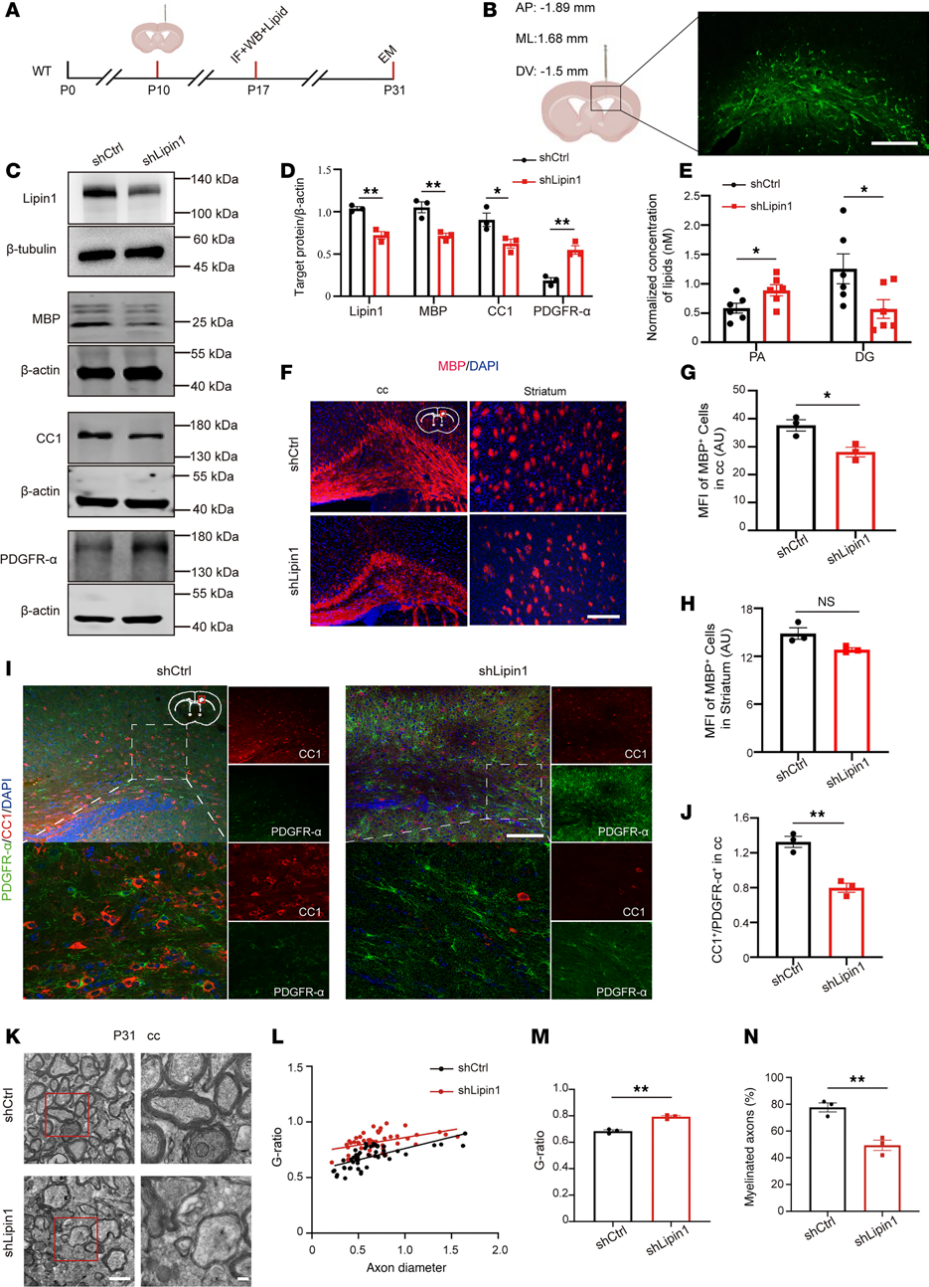
Reduced Lipin1 expression in the corpus callosum impairs OPC differentiation and myelination. (**A**) Experimental diagram. (**B**) Stereotaxic injection coordinates and infection area map of corpus callosum. Scale bar: 100 μm. (**C** and **D**) Western blot and statistical analysis of Lipin1, MBP, CC1, and PDGFR-α levels in the corpus callosum of C57BL/6J mice 7 days after stereotaxic injection of lentivirus (*n* = 3). Representative of 3 independent experiments. (**E**) PA and DG levels in the brain tissue of mice from both groups (*n* = 6). (**F**–**H**) MBP levels in the corpus callosum (cc) and striatum of C57BL/6J mice 7 days after stereotactic lentivirus injection were detected by immunofluorescent staining and statistically analyzed. Scale bar: 100 μm. (**I** and **J**) Statistical analysis of the CC1^+^/PDGFR-α^+^ (red/green) cell ratio and positive cells in the corpus callosum of C57BL/6J mice 7 days after stereotactic lentivirus injection was performed using immunofluorescent staining. Scale bars: 100 μm (low magnification) and 25 μm (high magnification). *n* = 3. (**K**) Two sets of TEM images of the myelin sheath in the corpus callosum. The high-magnification images are zoomed-in views of the red boxes in the low-magnification images. Scale bars: 1 μm (low magnification) and 200 nm (high magnification). (**L** and **M**) G-ratio distribution chart and statistical chart. (**N**) Percentage plot showing the number of myelinated axons. The number of myelinated axons evaluated was *n* = 50 per mouse, with 3 mice per group. Statistics: 2-tailed Student’s *t* test (**D**, **E**, **G**, **H**, **J**, **M**, and **N**). The results are expressed as mean ± SEM. *P < 0.05; **P < 0.01.

To investigate the effect of Lipin1 on myelination, Lipin1-knockdown lentivirus was injected into the corpus callosum (Figure 3A). IF imaging confirmed successful transfection (Figure 3B), and WB analysis validated effective Lipin1 knockdown (Figure 3, C and D). Knockdown of Lipin1 increased PA levels and decreased DG levels (Figure 3E). Differentiation of OPCs was impaired, as evidenced by significantly reduced MBP and CC1 (known as adenomatous polyposis coli protein) levels and increased PDGFR-α levels in the shLipin1 group (Figure 3, C and D). IF analysis further confirmed reduced MBP levels in the corpus callosum of the shLipin1 group (Figure 3, F–H) and a lower CC1^+^/PDGFR-α^+^ cell ratio (Figure 3, I and J). TEM analysis showed thinner myelin (Figure 3K) and a higher G-ratio in the shLipin1 group (Figure 3, L and M), along with fewer myelinated axons (Figure 3N). These results indicate that downregulation of Lipin1 in the corpus callosum impairs OPC differentiation and myelination.

Behavioral tests demonstrated that Lipin1 downregulation caused impaired motor and cognitive functions. The shLipin1 group showed reduced activity and speed in the open field test (Supplemental Figure 2, A–C), longer stance and swing durations in gait analysis (Supplemental Figure 2, D–F), and shorter running times on the rotarod (Supplemental Figure 2G). Spatial memory was also compromised, with longer latency and fewer platform crossings in the MWM (Supplemental Figure 2, H–K), and fewer entries and shorter stay durations in the novel arm of the Y-maze (Supplemental Figure 2, L and M). Previous studies have shown that structural damage to the corpus callosum leads to deficits in locomotion, spatial learning, and memory (23), supporting the functional relevance of our findings.

**Figure 4:**
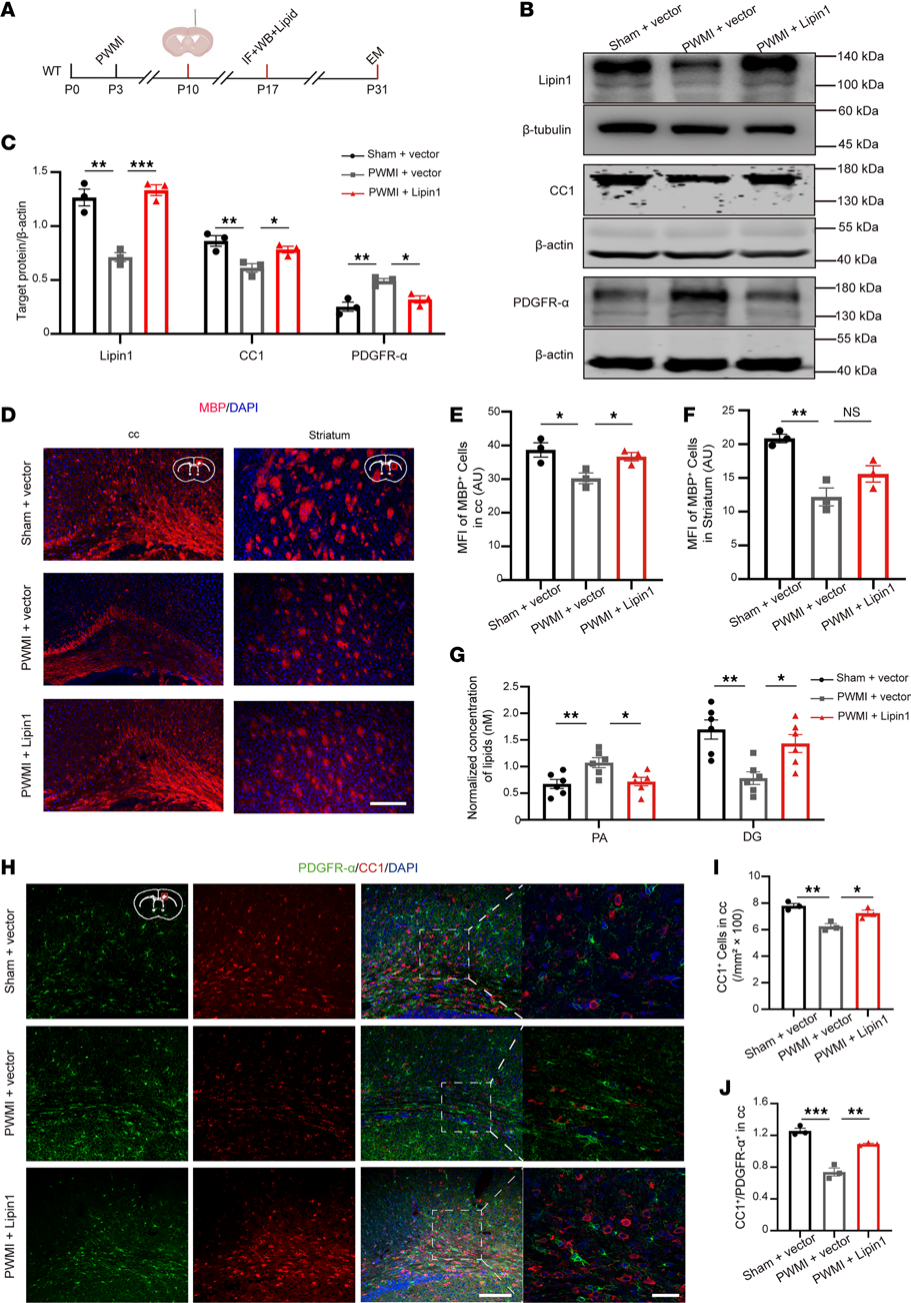
Overexpression of Lipin1 in the corpus callosum promotes OPC differentiation and myelination in PWMI mice. (**A**) Experimental diagram. (**B** and **C**) Western blot analysis of Lipin1, CC1, and PDGFR-α levels in the corpus callosum of mice 7 days after stereotaxic injection of lentivirus, with statistical analysis (*n* = 3). Representative of 3 independent experiments. (**D**) PA and DG levels in the brain tissue of mice from both groups (*n* = 6). (**E**–**G**) Immunofluorescent staining showing MBP levels in the corpus callosum and striatum of the 3 groups of mice, with statistical analysis (*n* = 3). Scale bar: 100 μm. (**H**–**J**) Immunofluorescence analysis of the number of CC1^+^ (red) and PDGFR-α^+^ (green) cells in the corpus callosum 7 days after lentiviral injection, and the ratio of positive cells, with statistical analysis. The high-magnification images are zoomed-in views of the white dashed boxes in the low-magnification images. Scale bars: 100 μm (low magnification) and 25 μm (high magnification). *n* = 3. Statistics: 1-way ANOVA with LSD post hoc analysis (**C**, **E**–**G**, **I**, and **J**). The results are expressed as mean ± SEM. *P < 0.05; **P < 0.01; ***P < 0.001. cc, corpus callosum.

To confirm the functional role of Lipin1, we overexpressed Lipin1 in the corpus callosum of PWMI mice (Figure 4A). WB and IF analyses showed restoration of CC1 and MBP levels and normalization of PDGFR-α levels (Figure 4, B–F and H–J). Additionally, chemical fluorescence analysis revealed correction of PA/DG metabolic imbalance (Figure 4G). TEM confirmed thicker myelin and a lower G-ratio in Lipin1-overexpressing PWMI mice (Supplemental Figure 3, A and B). Behaviorally, Lipin1 overexpression improved motor performance (open field, gait analysis, rotarod) and enhanced spatial memory (MWM, Y-maze) (Supplemental Figure 3, C–O). These results indicate that restoring Lipin1 expression alleviates both myelination deficits and behavioral impairments in PWMI mice.

### Reduced NAT10 expression in OPCs of PWMI mice.

**Figure 5:**
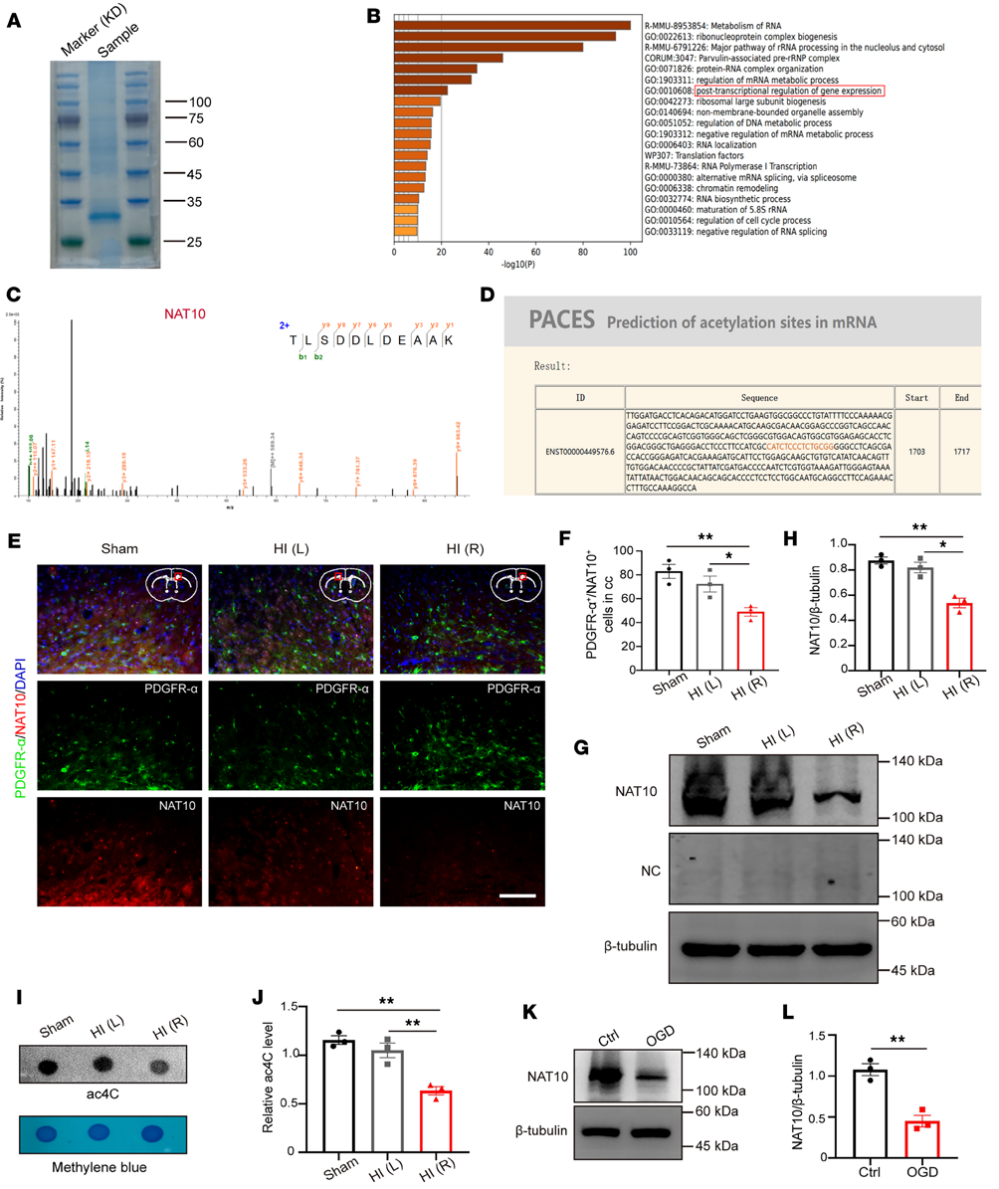
Reduced NAT10 expression in OPCs of PWMI mice. (**A**) Mass spectrometry results analysis and screening of Lipin1 posttranslational regulatory proteins. (**B**) GO enrichment analysis of Lipin1 mRNA binding protein pool. (**C**) Secondary mass spectrum of NAT10. (**D**) Prediction of potential ac4C sites on Lipin1 mRNA by PACES. (**E** and **F**) Immunofluorescent staining to detect and analyze the number of PDGFR-α^+^/NAT10^+^ (green/red) double-positive cells in the corpus callosum (cc) of mice. Scale bar: 50 μm. (**G** and **H**) Western blot analysis of NAT10 levels in the corpus callosum of mice and statistical analysis. NC, negative control (sample incubated with secondary antibody only, no primary antibody), confirming the specificity of the NAT10 signal. Representative of 3 independent experiments. (**I** and **J**) Dot blot analysis of RNA ac4C modification levels in the corpus callosum region and statistical analysis. Representative of 3 independent experiments. (**K** and **L**) Western blot analysis of NAT10 levels in primary cultured mouse OPCs after OGD and statistical analysis. *n* = 3. Statistics: 1-way ANOVA with LSD post hoc analysis (**F**, **H**, and **J**) and 2-tailed Student’s *t* test (**L**). The results are expressed as mean ± SEM. *P < 0.05; **P < 0.01.

To investigate the mechanisms regulating Lipin1 expression in PWMI mice, we performed RNA pull-down assays in primary cultured OPCs to identify proteins interacting with Lipin1 mRNA (Figure 5A). Gene Ontology (GO) enrichment analysis revealed that NAT10, an acetyltransferase involved in mRNA ac4C modification, was enriched in the “post-transcriptional regulation of gene expression” pathway (Figure 5B and Supplemental Table 1). Mass spectrometry analysis further confirmed the presence of NAT10 in the pull-down complexes (Figure 5C). We further predicted potential acetylation sites on Lipin1 mRNA using the PACES tool (http://rnanut.net/paces/), which identified a highly conserved acetylation site (Figure 5D).

To assess the levels of NAT10 protein and RNA ac4C modification in the corpus callosum of PWMI mice, tissues were collected 14 days after modeling for WB, IF, and dot blot experiments. IF results showed a significant reduction in the number of PDGFR-α^+^/NAT10^+^ double-positive cells in the corpus callosum of the HI (R) side compared with the Sham group and the HI (L) side (Figure 5, E and F). Consistently, WB analysis demonstrated markedly decreased NAT10 protein levels in the HI (R) corpus callosum (Figure 5, G and H). To confirm antibody specificity, a negative control in WB (secondary antibody only, no primary antibody) was performed, which showed no visible bands, indicating the specificity of the NAT10 signal (Figure 5G). Dot blot analysis revealed a significant reduction in RNA ac4C modification levels in the corpus callosum of the HI (R) side compared with the Sham group and HI (L) side (Figure 5, I and J). Similarly, primary OPC cultures demonstrated decreased NAT10 levels after 1 hour of OGD and 6 hours of reoxygenation (Figure 5, K and L). These findings indicate that NAT10 and RNA ac4C modification are significantly reduced in PWMI, suggesting their involvement in the pathology.

### NAT10 regulates ac4C acetylation of Lipin1 mRNA in OPCs.

**Figure 6:**
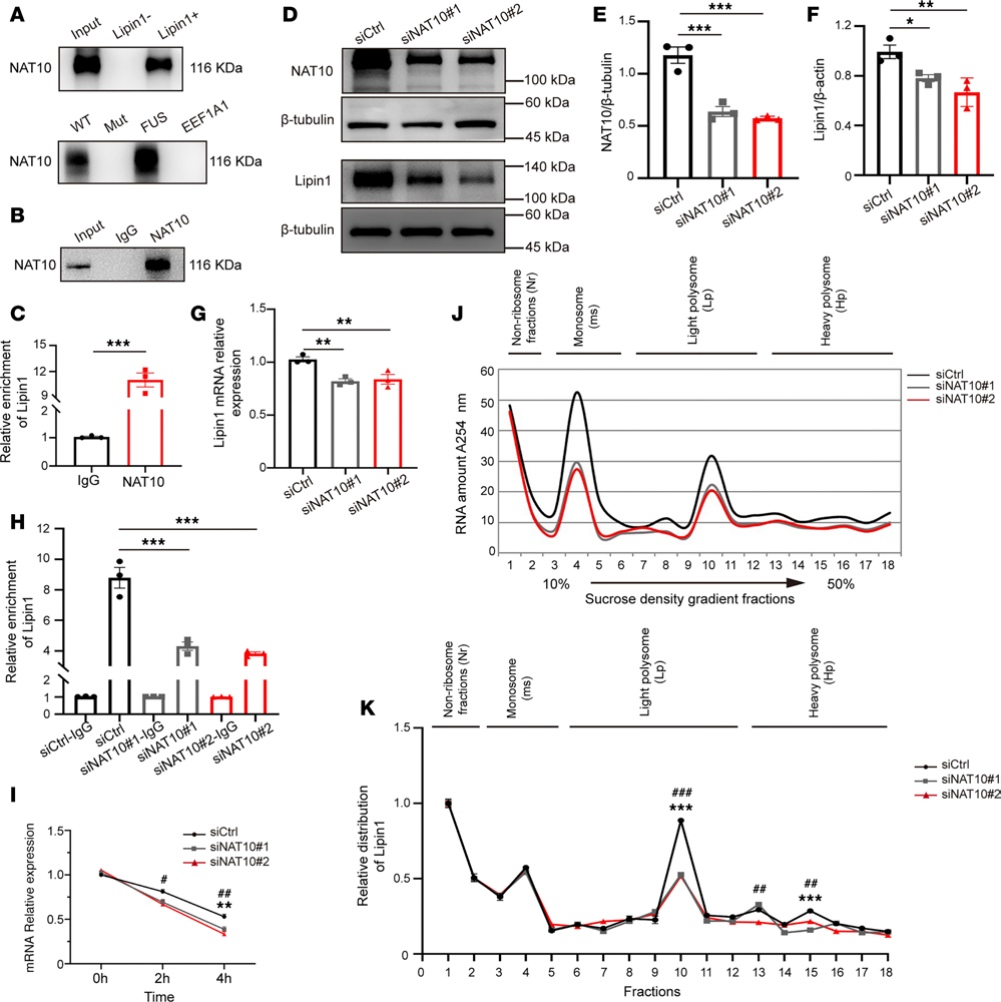
NAT10 regulated the ac4C acetylation of Lipin1 mRNA in OPCs. (**A**) RNA pull-down assay to verify the interaction between Lipin1 acetylation sites and NAT10 after constructing ac4C-site mutation plasmids. (**B** and **C**) In RIP experiments, Western blotting confirmed the expression of NAT10, and RT-qPCR measured the relative mRNA abundance of Lipin1. (**D**–**F**) Western blot analysis of NAT10 and Lipin1 protein levels in primary cultured OPCs and statistical analysis. Representative of 3 independent experiments. (**G**) RT-qPCR analysis of Lipin1 mRNA levels in primary cultured OPCs. (**H**) ac4C-RIP analysis of Lipin1 mRNA levels modified by ac4C in OPCs after NAT10 knockdown. (**I**) Cells treated with actinomycin D for 0, 2, and 4 hours to inhibit total RNA transcription, and RT-qPCR analysis of relative Lipin1 mRNA expression in mice. *siCtrl vs. siNAT10-1; ^#^siCtrl vs. siNAT10-2. (**J** and **K**) Sucrose density gradient centrifugation assay to validate the regulation of Lipin1 translation efficiency by NAT10. The position of mRNA in the sucrose gradient reflects its translation status: mRNA coprecipitating with ribonucleoproteins (RNPs) or ribosomal subunits indicates lack of translation, while coprecipitation with polysomes suggests active translation. Statistics: 2-tailed Student’s *t* test (**C**), 1-way ANOVA with LSD post hoc analysis (**E**–**H** and **K**), and 2-way ANOVA with Tukey’s multiple-comparison test (**I**). *siCtrl vs. siNAT10-1; ^#^siCtrl vs. siNAT10-2. *n* = 3. Results are presented as mean ± SEM. *P < 0.05, **P < 0.01, ***P < 0.001; ^##^*P* < 0.01, ^###^*P* < 0.001.

Given the acetyltransferase activity of NAT10, we next examined whether NAT10 regulates Lipin1 expression via mRNA acetylation. Based on PACES predictions, we identified a conserved acetylation site on Lipin1 mRNA and constructed a mutated Lipin1 plasmid (Mut) by replacing TCCCT with AGGGA. RNA pull-down assays showed that NAT10 directly binds to this acetylation site but the interaction was abolished upon mutation (Figure 6A), suggesting that NAT10 specifically targets this site. RNA immunoprecipitation (RIP) followed by reverse transcription quantitative real-time PCR (RT-qPCR) confirmed a significant enrichment of Lipin1 mRNA in NAT10-immunoprecipitated complexes compared with IgG controls (Figure 6, B and C). To further validate the functional role of NAT10, we knocked down NAT10 in OPCs using siRNA (siNAT10). Both WB and RT-qPCR results showed a marked reduction in Lipin1 expression (Figure 6, D–G), highlighting NAT10’s positive regulation of Lipin1. Furthermore, ac4C-RIP experiments in NAT10-knockdown OPCs showed a decrease in ac4C-modified Lipin1 mRNA levels (Figure 6H). Moreover, actinomycin D chase experiments demonstrated that Lipin1 mRNA stability was significantly impaired upon NAT10 knockdown (Figure 6I), and sucrose density gradient centrifugation revealed fewer polysome complexes and decreased polysomal Lipin1 mRNA (Figure 6, J and K). Together, these results suggest that NAT10 may regulate Lipin1 expression by promoting ac4C modification, thereby enhancing mRNA stability and translation efficiency.

### NAT10 downregulation inhibits OPC differentiation by suppressing Lipin1 expression in vitro.

**Figure 7:**
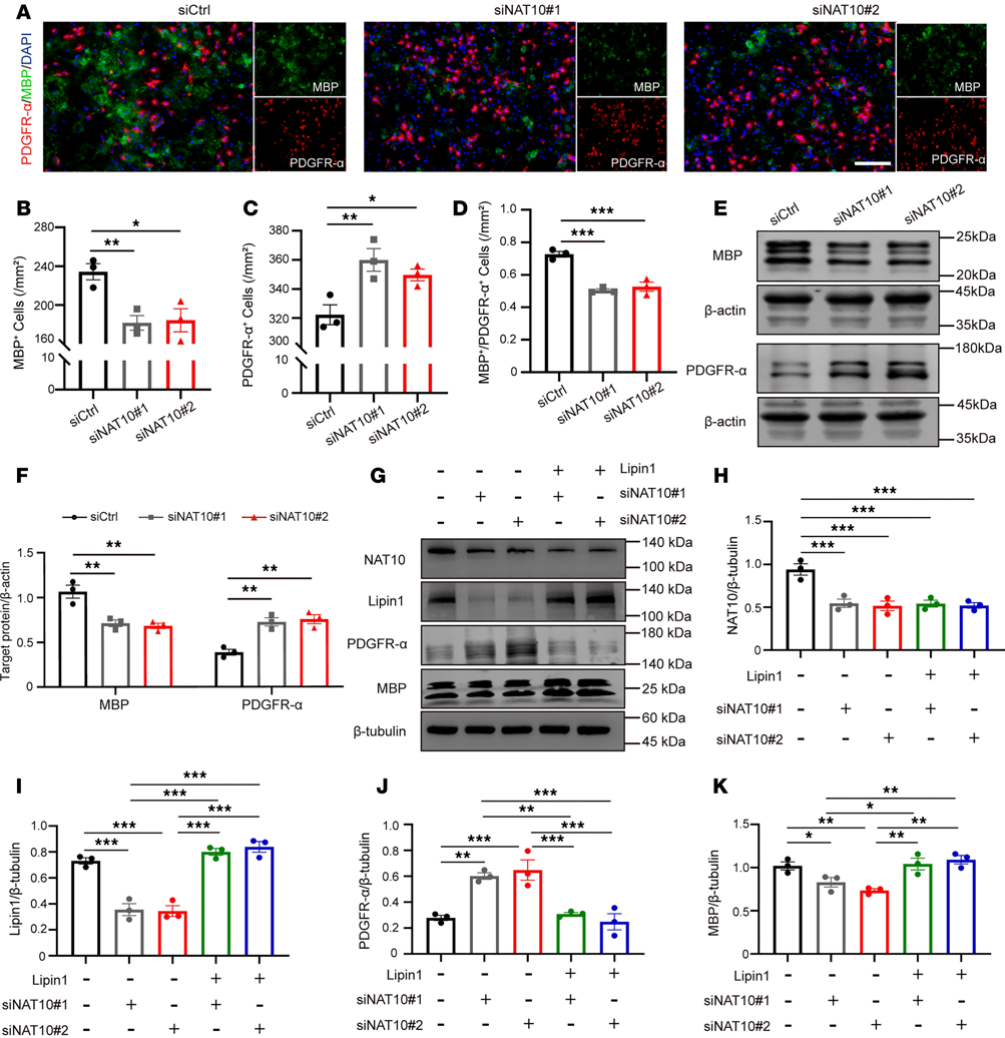
Downregulation of NAT10 inhibits OPC differentiation by suppressing Lipin1 expression in vitro. (**A**) Immunofluorescent staining to detect the number of MBP^+^ (green) and PDGFR-α^+^ (red) cells during differentiation in primary mouse OPCs with NAT10 knockdown. Scale bar: 100 μm. (**B**–**D**) Statistical analysis of the number of MBP^+^ cells and PDGFR-α^+^ cells, and the MBP^+^/PDGFR-α^+^ cell ratio in primary mouse OPCs. (**E** and **F**) Western blot analysis of MBP and PDGFR-α protein levels in primary cultured OPCs and statistical analysis. Representative of 3 independent experiments. (**G**–**K**) Western blot analysis of NAT10, Lipin1, PDGFR-α, and MBP protein levels in OPCs under different treatments and statistical analysis. Representative of 3 independent experiments. Statistics: 1-way ANOVA with LSD post hoc analysis (**B**–**D** and **H**–**K**) and 1-way ANOVA with Dunnett’s test (**F**). *n* = 3. Results are presented as mean ± SEM. *P < 0.05; **P < 0.01; ***P < 0.001.

To determine the biological impact of NAT10 on OPC function, we evaluated OPC differentiation following NAT10 knockdown. IF results revealed a significant decrease in MBP^+^ cells and an increase in PDGFR-α^+^ cells in the siNAT10 group compared with the siCtrl group (Figure 7, A–D). WB analysis confirmed reduced MBP levels and elevated PDGFR-α expression following NAT10 knockdown (Figure 7, E and F). To assess whether Lipin1 mediates this effect, we performed a rescue experiment by overexpressing Lipin1 in NAT10-deficient OPCs. Overexpression of Lipin1 significantly reversed the changes in PDGFR-α and MBP levels induced by NAT10 knockdown (Figure 7, G–K). These findings suggest that NAT10 promotes OPC differentiation at least in part through the regulation of Lipin1 expression.

## Discussion

Recent studies have emphasized the crucial role of lipid metabolic balance in OPC differentiation and myelination development. Specifically, the accumulation of 8,9-unsaturated sterol intermediates in the cholesterol synthesis pathway has been identified as a central mechanism that promotes OPC differentiation and maturation (24). During development, OPCs progressively reorganize their lipid metabolism and alter membrane lipid composition, significantly increasing the biosynthesis of cholesterol and galactolipids while reducing the relative amounts of phospholipids and proteins (12). Furthermore, targeted metabolomics studies of postmortem brain tissue from patients with CP have revealed significant dysregulation in glycerophospholipid metabolism (25). Glycerophospholipids are mainly synthesized within the brain and not only serve as structural components of cell membranes but also act as second messengers and precursors, participating in membrane fusion, apoptosis, and the regulation of membrane-bound enzymes and ion channels (26–28). Lipin1 protein plays a central role in the penultimate step of the glycerol phosphate pathway and catalyzes the conversion of PA to DG (29). The molecular and cellular roles of Lipin1 in OL development and regeneration in vivo have yet to be investigated.

Lipin1-deficient mice exhibit peripheral demyelination, hyperlipidemia, and lipodystrophy, highlighting its role in lipid metabolism and myelin formation (30, 31). Our study found that Lipin1 expression in OPCs is significantly downregulated after HI injury, accompanied by elevated PA and reduced DG in the brain. Lipin1 expression in the corpus callosum decreases 14 days after HI but increases later, potentially regulated by lipid metabolites activating peroxisome proliferator-activated receptor α (PPARα) (32). We showed that Lipin1 downregulation impairs OPC differentiation and myelination, while its overexpression improves these processes and motor function in PWMI mice. The corpus callosum is the largest white matter structure in the brain and plays a critical role in interhemispheric information transfer. Its proper myelination is essential for coordinated motor function and cognitive processes (33). Clinical and preclinical studies have shown that damage to the corpus callosum is associated with deficits in locomotion, learning, and memory (34). The observed behavioral abnormalities in our study — including impaired motor coordination and spatial memory — are likely attributable to hypomyelination of the corpus callosum. This highlights the functional importance of Lipin1 in maintaining the structural and physiological integrity of this key brain region.

Our study provides what we believe to be the first evidence implicating Lipin1 in OPC differentiation. As a PAP, Lipin1 regulates glycerophospholipid metabolism and triglyceride (TG) synthesis by converting PA to DG. In the glycerol phosphate pathway, the final and only committed step is to form a TG by covalently joining a fatty acyl-CoA and a DG molecule (35). Lipin1 deficiency has been shown to promote axonal regeneration after injury by modulating TG hydrolysis and phospholipid synthesis (36). Additionally, reduced Lipin1 levels in hyperglycemia disrupt hippocampal mitochondrial dynamics and impair cognitive function in diabetic encephalopathy (37). These findings emphasize Lipin1’s critical role in disease pathology. Moreover, DG, via the Kennedy pathway, produces phosphatidylethanolamine and phosphatidylcholine, which are essential for myelin integrity (15, 22), and phosphatidylethanolamine deficiency is linked to myelin dysfunction (38). PA and DG also act as second messengers, activating protein kinase C (PKC) and mechanistic target of rapamycin (mTOR), which regulate myelination (39–41). Besides its enzymatic activity, Lipin1 also regulates gene expression by translocating to the nucleus and interacting with transcription factors (16), highlighting its role in cellular homeostasis and lipid metabolism.

To investigate the mechanisms behind Lipin1 downregulation in PWMI, we used RNA pull-down to isolate proteins binding to Lipin1 mRNA from primary OPCs, followed by mass spectrometry analysis. We focused on NAT10 as a key modulator of these processes because it is the only acetyltransferase known to catalyze the addition of ac4C to RNA substrates, with acetyl-CoA serving as the acetyl donor (20, 42). ac4C modifications are common in human mRNA, ensuring accurate codon reading during translation (43). Using the PACES database, we predicted acetylation sites on Lipin1 mRNA, and RIP and RNA pull-down experiments in OPCs confirmed that NAT10 mediates its ac4C modification by directly binding to the mRNA. Arango et al. utilized transcriptome-wide techniques to examine the distribution and functional impact of ac4C in mRNA, revealing that NAT10-mediated ac4C modification enhances the stability and translation efficiency of target mRNAs (19). In our study, we found that HI injury decreases NAT10 levels in OPCs, reducing Lipin1 mRNA stability and translation, thereby inhibiting Lipin1 expression. The mechanisms underlying NAT10 downregulation after HI injury remain to be fully elucidated. However, we speculate that the HI environment may suppress NAT10 expression through several pathways. First, HI-induced oxidative stress and inflammatory signaling (e.g., via TNF-α or IL-1β) may inhibit NAT10 transcription or promote its degradation, as has been shown for other RNA-modifying enzymes under stress conditions (44). Second, metabolic stress such as reduced acetyl-CoA availability — a key substrate for NAT10’s enzymatic activity — could impair both its function and expression (45). Third, epigenetic modifications such as promoter methylation or histone deacetylation may also suppress NAT10 expression in response to HI-induced cellular reprogramming (46). Further studies are needed to explore these mechanisms.

While most studies on NAT10 have focused on its role in cancer, where it regulates gene expression to promote tumor progression and resistance to therapy (47–49), lipid metabolism dysregulation has emerged as one of the most prominent metabolic changes associated with cancer (50). NAT10-mediated ac4C modification stabilizes lipid metabolism–related genes, including ELOVL6, ACSLs and ACAT1 (51). Our findings suggest that NAT10, through its regulation of Lipin1, contributes to myelination disruption in PWMI, implicating it in the disorder’s pathogenesis. NAT10-mediated ac4C modification has been linked to several human diseases, including depression (21), myocardial infarction (52), interstitial cystitis (53), and acquired immune deficiency syndrome (AIDS) (54). Inhibition of NAT10 can improve nuclear shape, repair DNA damage, and reverse cell proliferation defects, offering potential treatment for Hutchinson-Gilford progeria syndrome (55). Yang et al. found that overexpressing NAT10 in ovariectomized mice reversed bone loss (56), further supporting NAT10 as a promising therapeutic target for various diseases. Our study connects NAT10-mediated ac4C modification to myelination defects in PWMI, suggesting a potential therapeutic strategy for addressing this disorder.

Our study has limitations, including the need for further exploration of lipid metabolic pathways and OPC differentiation. Detailed lipidomic profiling of OPCs and myelin may reveal additional contributors to myelination defects. Moreover, using a single PWMI animal model limits clinical relevance. Future research with preterm birth models or iPSC-derived OPCs is needed for validation. In conclusion, our study provides further mechanistic insights into the molecular mechanisms underlying myelination defects in PWMI, not only broadening our understanding of lipid metabolism in OPC biology but also suggesting that targeting Lipin1 and NAT10 could offer potential therapeutic avenues for treating myelination defects in PWMI.

## Methods

### Sex as a biological variable

Both male and female neonatal mice were used without sex-based distinction. No sex-related differences were observed, and the results are considered relevant to both sexes.

### Animals

Specific pathogen–free–grade wild-type C57BL/6J mice were purchased from the Animal Center of Xuzhou Medical University. After 1 week of adaptation to the new environment, the mice were mated at the onset of puberty (males at 8 weeks of age, females at 6 weeks of age), with 1 male and 2 females per cage. Female mice were checked daily for the presence of a vaginal plug to determine the start of pregnancy, after which males were promptly separated. The day of birth was considered day 0, and subsequent days were numbered accordingly. All animals were housed in individually ventilated cage mouse housing systems with free access to food and water. The lighting cycle followed a 12-hour light/dark schedule, with room temperature maintained at 20°C–22°C and relative humidity at 50%–60%. The mouse feed was obtained from the Animal Experiment Center of Xuzhou Medical University, and drinking water was replaced every 3 days.

### Development of the PWMI mouse model

Unsexed 3-day-old C57BL/6J pups were anesthetized using isoflurane (3% for induction and 1.5% for maintenance). Upon achieving anesthesia, the pups were placed in a supine position on a thermostatically controlled surgical table maintained at 37°C, and their heads and limbs were gently but securely immobilized. Under a stereomicroscope (Olympus SZ61), the right common carotid artery of each pup was exposed, carefully isolated from surrounding nerves and veins, and ligated with 6-0 surgical silk (Ethicon). The fat and fascia layers were then closed sequentially, and the incision was sutured. The total duration of the surgical procedure was limited to 5 minutes. Following the surgery, the pups were placed in an anoxic chamber for 1.5 hours, which contained a gas mixture of 8% O_2_ and 92% N_2_. Oxygen and nitrogen concentrations were monitored in real time using a gas analyzer (BioSpherix ProOx 110). After the anoxia period, the pups were returned to their mother’s cage. Sham-operated animals underwent the same procedure, except the carotid artery was not ligated. All surgical instruments were sterilized before use, and all procedures were performed in accordance with institutional guidelines for animal care and use.

### IF staining

The brain sections (thickness: 20 μm, prepared by freezing microtome) were incubated in 5% bovine serum albumin (BSA) (VICMED, VIC018) and 0.3% Triton X-100 (VICMED, VIC398) in 0.01 mol/L PBS for 30 minutes at 37°C to block nonspecific reactions. For cell samples, 200 μL of 4% paraformaldehyde (Solarbio, P1110) was added to each well of a 24-well plate and incubated at room temperature for 20 minutes. Then, the samples were washed with PBS for 5 minutes, repeating the wash 3 times. Membrane permeabilization and blocking were performed simultaneously with 0.3% Triton X-100 and 5% BSA in PBS for 30 minutes at room temperature. Sections or cells were incubated overnight at 4°C with primary antibodies anti-MBP (Santa Cruz Biotechnology, sc-376995; 1:200), anti–PDGFR-α (Abcam, ab203491; 1:300), anti-Olig2 (Millipore, MABN50; 1:500), anti-Lipin1 (Abcam, ab181389; 1:200), anti-CC1 (Thermo Fisher Scientific, PA5-30580; 1:200), and anti-NAT10 (Proteintech, 13365-1-AP; 1:500). After primary antibody incubation, sections and cells were washed 3 times with PBS (5 minutes each), and then incubated with fluorescently labeled secondary antibodies (goat anti–mouse IgG Alexa Fluor 488, goat anti–rabbit IgG Alexa Fluor 594; both from Abcam; 1:500) for 1 hour at room temperature in the dark. After secondary antibody incubation, sections and cells were washed with PBS 3 times. Nuclei were counterstained with DAPI (Solarbio, C0060) for 5 minutes at room temperature, followed by final washes. Imaging was performed with a fluorescence microscope (Olympus BX53, equipped with DP80 camera and CellSens Dimension software). All images were acquired with consistent exposure times and gain settings. For each staining set, at least 3 independent biological replicates were performed.

### WB analysis

The brain tissue and cells samples were rapidly isolated to perform WB detection. WB analysis was performed as previously described (57). Total proteins were extracted by ultrasonically homogenizing brain tissues in ice-cold RIPA lysis buffer (Beyotime, P0013B) supplemented with 1% PMSF (Beyotime, ST506). For cytoplasmic protein isolation, a cytoplasmic protein extraction kit (Beyotime, P0027) was utilized according to the manufacturer’s guidelines. Protein concentrations were quantified using the BCA Protein Assay Kit (Beyotime, P0012). An equivalent amount of protein (20–30 μg) from each sample was mixed with 5× SDS loading buffer, denatured at 95°C for 5 minutes, and separated in 10% SDS-polyacrylamide gels. Subsequently, proteins were transferred onto 0.22 μm PVDF membranes (Millipore, IPVH00010) by wet electrotransfer. Following transfer, membranes were blocked in PBST containing 5% skim milk for 1 hour at room temperature and incubated overnight at 4°C with the following primary antibodies: anti-MBP (Santa Cruz Biotechnology, sc-376995; 1:1000), anti–PDGFR-α (Abcam, ab203491; 1:1000), anti-Lipin1 (Abcam, ab181389; 1:1000), anti-CC1 (Thermo Fisher Scientific, PA5-30580; 1:1000), anti-NAT10 (Proteintech, 13365-1-AP; 1:1000), and anti–β-actin (Proteintech, 66009-1-Ig; 1:5000). As a negative control, membranes were also incubated with secondary antibodies only, omitting the primary antibodies, to assess nonspecific binding. After washing, membranes were incubated for 2 hours at room temperature with either IRDye 680RD Goat anti-Rabbit IgG (LI-COR, 926–68071; 1:10,000) or IRDye 800CW Goat anti-Mouse IgG (LI-COR, 926–32210; 1:20,000) secondary antibodies. Protein bands were visualized and analyzed using an Odyssey infrared imaging system (LI-COR Biosciences). Band intensities were quantified using ImageJ software version 1.8.0 (NIH). All experiments were independently repeated at least 3 times.

### TEM

For TEM analysis, mouse brains were perfused with an electron microscope fixative (containing 2% glutaraldehyde and 4% paraformaldehyde), and the corpus callosum was isolated on ice. A tissue block (~1 mm³) was cut from the corpus callosum, maintaining consistent sampling across groups. The tissue was then fixed overnight in 2.5% glutaraldehyde at 4°C, followed by PBS washes (10 minutes, repeated 3 times). Afterward, the samples were fixed in 1% osmium tetroxide in phosphate buffer at room temperature for 2 hours, followed by washing in ddH_2_O (10 minutes, repeated 3 times). The samples were dehydrated through a graded ethanol series (30%, 50%, 70%, 80%, 90%, 95%, 100%) and then transitioned into 100% acetone. Dehydration was followed by infiltration with a 1:1 mixture of acetone and 618 epoxy resin for 2–4 hours, then with a 2:1 acetone/resin mixture overnight. The samples were subsequently embedded in pure resin for 5–8 hours (3 exchanges), placed into embedding molds, and polymerized overnight at 37°C. After polymerization, the samples were sectioned into 50-nm-thick slices, stained with lead citrate for 15 minutes, and washed with ddH_2_O. Sections were dried at room temperature and observed under TEM. High-magnification images (×6000 magnification) were captured, and the G-ratio was calculated as the ratio of the inner diameter to the total outer diameter of the myelinated axon. Three samples per group were analyzed.

### Behavioral experiment methodology

#### MWM assessment.

The MWM was used to evaluate spatial location learning and memory as described in previous studies (57). The MWM test was performed using a circular pool (diameter 1.2 m, height 40 cm) filled with water maintained at 25°C, equipped with a movable platform, temperature control, and a video tracking system (ANY-maze, Stoelting Co.). Each group included 8 mice, acclimated to the testing room 2–3 hours before testing, which was conducted between 9:00 am and 12:00 pm. The platform (8 cm diameter) was submerged 0.5–1 cm below the water surface. To enhance tracking, powdered milk was added, and visual cues were placed around the pool, which was divided into quadrants in the ANY-maze software. During training, mice were placed on the platform for 30 seconds, then released from random quadrants facing the wall to search for the platform. Each mouse underwent 2 sessions daily (2 trials per session) for 4 consecutive days. If a mouse failed to find the platform within 60 seconds, it was guided to the platform and allowed to remain for 30 seconds. On the fifth day, a probe trial was conducted by removing the platform; mice were released from the opposite quadrant, and swimming behavior was recorded for 60 seconds. The number of platform crossings, time spent, and entries into each quadrant were analyzed automatically by ANY-maze. All data were recorded using a computerized video system (Ethovision 3.1, Noldus Instruments).

#### Open field test.

The open field test was conducted using a 50 cm × 50 cm × 30 cm arena and the ANY-maze automated tracking system. Mice were acclimated for 2 hours before testing. Each mouse was placed in the center of the arena for 5 minutes of free exploration while the system recorded total distance traveled, average speed, and time spent in the central and peripheral zones. After each trial, the arena was cleaned with 75% ethanol and paper towels.

#### Y-maze.

In the Y-maze test, animals were first placed in the maze with 1 arm closed for 3 minutes of exploration in the remaining 2 arms. After 4 hours, a recall phase was conducted where all arms were opened, allowing the animal to explore freely for 3 minutes. The number of entries, exploration time, and path length in each arm were recorded to assess memory and spontaneous alternation behavior.

#### Rotarod test.

In the rotarod test, animals underwent a training phase where they start at 4 rpm for 1 minute, then progress to 10 rpm for 2 minutes, and finally 30 rpm for 3 minutes, with 15-minute rest periods between each stage. After a 24-hour rest, the formal test began by placing the mouse on a rotating rod (accelerated to 20 rpm) and recording the latency to fall. If a mouse fell, it was promptly removed and returned to its cage. After 5 minutes, any remaining mice were moved to their cage. The maximum score is 300 seconds, with 3 trials conducted per mouse, and the average latency was used for statistical analysis.

#### Gait analysis.

In the gait analysis, mice were acclimated to the testing environment 2 days prior using the VisuGait system (Zhenghua Biological Instrument and Equipment Co., Ltd.). During the training phase, mice were placed in the gait tunnel with a cage mate for 5–10 minutes to familiarize themselves with the environment, without testing. Afterward, they were individually placed at one end of the tunnel, with the other end blocked, and allowed to run twice daily for 1 week. In the formal testing phase, the tunnel exit was blocked, and the mice were placed at the entrance. Data were recorded as the mouse passed through the camera zone, with each mouse tested 5 times. The gait data, including stance duration, swing duration, and propulsion time, were averaged and analyzed for statistical significance.

### Measurement of PA and DG

PA was measured by using the PicoProbe Phosphatidic Acid Assay Kit (Abcam, ab273335) according to the manufacturer’s instructions. Briefly, brain tissue was homogenized in the provided PA assay buffer, and lipid extraction was performed according to the protocol. The lipids were solubilized in a 5% Triton X-100 solution. Parallel preparations of “Sample” and “Sample background control” were made. A standard curve was created using the PA standard solution. The converter mix was added exclusively to the sample and standard wells. After incubation at 45°C for 1 hour, reaction mix was introduced into all wells and incubated at 37°C for 30 minutes. Fluorescence was measured with an excitation/emission of 535/587 nm. The PA concentration was normalized based on the recorded values.

DG was measured by using the Diacylglycerol Assay Kit (Abcam, ab242293) according to the manufacturer’s instructions. Brain tissue was homogenized and mixed with methanol, NaCl, and chloroform, followed by phase separation through centrifugation. The organic phase was washed twice with a pre-equilibrated upper phase solution, then dried under nitrogen flow and resuspended in assay buffer. For the assay, DG standards, samples, and blanks were prepared in a 96-well plate. A kinase mixture was added to half of the sample wells, and buffer to the other half, followed by incubation at 37°C for 2 hours. After transferring the mixture to a suitable plate, lipase solution was added, and incubation continued for 30 minutes. The detection enzyme mixture was then added, and fluorescence was measured at Ex/Em = 530–560 nm/585–595 nm after 10 minutes of incubation at room temperature. Results were calculated by subtracting background values and generating a standard curve for further analysis.

### RT-qPCR

Total RNA was extracted using TRIzol reagent (Invitrogen, 15596018) following the manufacturer’s protocol. RNA concentration and purity were assessed using a NanoDrop 2000 spectrophotometer (Thermo Fisher Scientific). Reverse transcription was carried out with 1 μg of total RNA using the HiScript III 1st Strand cDNA Synthesis Kit (Vazyme, R312-01) according to the manufacturer’s instructions. qPCR was performed using ChamQ Universal SYBR qPCR Master Mix (Vazyme, Q711-02) on a LightCycler R480 PCR System (Roche) with the following cycling conditions: 95°C for 30 seconds, followed by 40 cycles of 95°C for 10 seconds and 60°C for 30 seconds (annealing temperature). Melting curve analysis was conducted to verify the specificity of amplification. Primer sequences, expected amplicon sizes (in bp), and GenBank accession numbers are provided in Supplemental Table 2. Relative gene expression was calculated using the 2^–ΔΔCt^ method, and Gapdh was used as the internal reference gene (housekeeping gene). All primer pairs were validated using NCBI Primer-BLAST (https://www.ncbi.nlm.nih.gov/tools/primer-blast/) to ensure specificity. No off-target amplification was observed, and representative screenshots of BLAST results are presented in Supplemental Figure 4.

### Virus packaging

Lipin1 overexpression and RNA interference lentiviruses and their respective blank vectors were obtained from Shanghai GeneChem Corporation. The titer of the lentiviruses was 2 × 10^9^ TU/mL. The sequences for Lipin1 shRNA and its blank vector are provided in Supplemental Table 3. These lentiviruses were administered in vivo via stereotaxic injection into the corpus callosum to modulate the expression of Lipin1.

### Stereotaxic injection

The stereotaxic injection procedure began by anesthetizing mice with an intraperitoneal injection of sodium pentobarbital (50 μg/g) and securing them on a stereotaxic frame, ensuring that the Bregma and Lambda points were level. After disinfecting the skin with povidone-iodine, an incision was made, and the periosteum was cleaned with 3% hydrogen peroxide to expose the Bregma point. A microsyringe was positioned so that the needle touched the Bregma, and the coordinates were recorded as the origin. The final injection coordinates were determined based on the Mouse Brain Atlas (AP: –1.89 mm, ML: 1.68 mm, DV: –1.5 mm). The virus was injected at 0.2 μL/min for 10 minutes, and the needle remained in place for another 10 minutes before being slowly withdrawn. The incision was sutured, and the mice were returned to their cages for recovery.

### Culture of primary OPCs

#### Isolation of mixed glial cells.

Newborn mice (0–2 days) were anesthetized on crushed ice, and after disinfection with 75% ethanol, brain tissues were quickly extracted and placed in prechilled DMEM/F-12 medium. The cortical tissues were isolated under a stereomicroscope, and the meninges and non-cortical regions were removed. The cortices were minced and transferred to a 15 mL centrifuge tube containing prechilled DMEM/F-12 medium. The tissue was gently triturated 10–15 times using a sterile Pasteur pipette, allowed to settle for 2 minutes, and the supernatant was transferred to a new sterile tube. This process was repeated until the brain tissues were dissociated into single-cell suspensions. The suspensions were centrifuged at 145*g* for 5 minutes, and the supernatant was discarded. The cell pellet was resuspended in a small volume of complete medium and filtered through a 40-μm nylon mesh. The filtered cells were seeded into poly-D-lysine–coated T25 flasks at a density of 1.0 × 10^6^ to 2.0 × 10^6^ cells/flask and cultured in DMEM/F-12 medium containing 10% FBS in a 5% CO_2_ incubator. Media were changed every 2 days, and after about 1 week, OPCs were isolated by shaking.

#### Purification and culture of OPCs.

For OPC purification, cells were detached using a non-trypsin cell dissociation solution and centrifuged. OPCs were plated at a density of 2 × 10^4^ cells/mL.

#### Induced differentiation of OPCs.

For passaging and differentiation, bFGF (10 ng/mL) was added to the medium to promote OPC proliferation. When cells reached confluence, they were passaged at a 1:2 ratio. To induce OPC differentiation, 15 nM triiodothyronine was added to the medium, and the supply of PDGF and bFGF was discontinued. The medium was replaced every 3 days, and cells were cultured for 5–10 days to induce maturation.

### siRNA transfection

The medium was replaced with 1.5 mL of serum-free medium in the culture dish. To prepare the transfection complex, 3 μL of siRNA was added to 250 μL of serum-free medium, mixed, and incubated for 5 minutes. In a separate tube, 6 μL of SilentFect Lipid Reagent (Bio-Rad) was mixed with 250 μL serum-free medium and incubated for 5 minutes. Afterward, the SilentFect mixture was combined with the siRNA solution and incubated for 20 minutes. The transfection mixture was added to the culture dish, ensuring even distribution, and incubated at 37°C in a 5% CO_2_ incubator. The medium was replaced after 6–8 hours, and protein or RNA was harvested 24–96 hours later for further analysis.

### RNA pull-down

Lipin1 probes with 3′ biotin labels were designed and synthesized by Guangzhou RiboBio Co., Ltd. Both Lipin1 sense and antisense probes were dissolved in nuclease-free water to a concentration of 100 μM. Prior to experiments, protease inhibitor cocktail (Beyotime, P1005) and RNase inhibitor (Vazyme, R411-01) were thawed at room temperature. Lysis buffer was freshly prepared by mixing WB/IP lysis buffer (Beyotime, P0013) with protease inhibitor cocktail at a 1:99 v/v ratio, and RNase inhibitor was added to a final concentration of 1 U/μL to prevent RNA degradation. A minimum of 20–30 million cells were digested with trypsin, collected, and lysed in 1 mL of lysis buffer on ice. After centrifuging at 17,000*g* for 15 minutes, the supernatant was collected, and 1 μL of either Lipin1 or antisense probe was added. The mixture was incubated on a rotator at 4°C for 6 hours or overnight, followed by the addition of 50 μL streptavidin magnetic beads (Thermo Fisher Scientific, 88817) for 3 hours. After magnetic separation, the beads were washed 3 times with lysis buffer and resuspended in 60 μL 2× SDS-PAGE buffer, and then heated at 100°C for 10 minutes for further analysis or storage.

### Mass spectrometry analysis

Mass spectrometry analysis was performed by Shanghai Zhongke Xin Life Biotechnology Co., Ltd. Protein samples were first reduced with 10 mM dithiothreitol (DTT) at 56°C for 1 hour and subsequently alkylated with 20 mM iodoacetamide at room temperature in the dark for 45 minutes. After reduction and alkylation, proteins were enzymatically digested with sequencing-grade modified trypsin at an enzyme-to-substrate ratio of 1:50 (w/w) at 37°C for 20 hours. After desalting and lyophilization, the digest was redissolved in 0.1% formic acid solution and stored at –20°C. For analysis, a mobile phase of 0.1% formic acid in water (A) and 0.1% formic acid in acetonitrile (B) was used. The peptides were loaded onto a C18 reversed-phase analytical column (Thermo Fisher Scientific, 75 μm × 250 mm, 2 μm particle size) and separated using a linear gradient of 5%–35% B over 90 minutes at a flow rate of 300 nL/min. Mass spectrometry was conducted in positive ion mode with a scan range of 300–1800 *m*/*z*, a resolution of 70,000 at 200 *m*/*z*, and an AGC target of 1 × 10^6^. After each full scan, 20 MS2 scans were acquired using HCD activation, with a collision energy of 27 eV. Data quality control was performed by monitoring the reproducibility of identified peptides across biological replicates and extracting specific marker peaks. The data were analyzed using Proteome Discoverer 2.5 (Thermo Fisher Scientific), and protein identification was achieved by searching the appropriate database.

### Dot blot assay

Total RNA was extracted from tissues using TRIzol reagent according to the manufacturer’s instructions. The RNA concentration was measured using a NanoDrop 2000 spectrophotometer and RNA integrity was confirmed by agarose gel electrophoresis. RNA was extracted from tissues and denatured by heating at 95°C for 3 minutes, then placed on ice. Two microliters of the denatured RNA was applied to a Hybond-N+ membrane (Sigma-Aldrich), crosslinked with UV light for 1 minute, and repeated once. The membrane was washed in washing buffer, blocked for 1 hour, and incubated overnight at 4°C with anti-ac4C antibody (Abcam, ab252215; 1:1000). After 3 washes with TBST (5 minutes each), the membrane was incubated with HRP-conjugated goat anti-rabbit IgG secondary antibody for 1 hour at room temperature. Finally, the membrane was washed and exposed to ECL Western Blotting Reagents (Thermo Fisher Scientific, 32106) for 5 minutes before imaging.

### RIP

Proteinase inhibitor cocktail (Sigma-Aldrich, P8340) and RNase inhibitor (Thermo Fisher Scientific, EN0531) were thawed at room temperature. WB and IP lysis buffers were prepared by mixing 1× PBS with 0.5% NP-40, 1 mM DTT, 1 mM EDTA, 1 mM PMSF, and 1× proteinase inhibitor cocktail. The RNase inhibitor was added to achieve a final concentration of 1 U/μL, ensuring the RNA was protected from degradation during the process. The WB and IP lysis buffers were mixed with the proteinase inhibitor cocktail in a 99:1 ratio, and RNase inhibitor was added to achieve a final concentration of 1 U/μL, serving as the lysis buffer. Cells were collected, centrifuged, and resuspended in 1 mL of the prepared lysis buffer. The lysates were subjected to repeated freeze-thaw cycles in liquid nitrogen to disrupt the cell membranes. Afterward, the lysates were centrifuged at 17,000*g* for 15 minutes at 4°C to remove cellular debris, and the supernatant was carefully collected. To capture RNA-protein complexes, the supernatant was incubated with 2 μg of specific antibody (anti-FLAG, Abcam, ab1253; or anti-Myc, Cell Signaling Technology, 2276) overnight at 4°C with gentle rotation. Protein A/G magnetic beads (Thermo Fisher Scientific, 88802) were then added to the lysate-antibody mixture and incubated for an additional 3 hours at 4°C. Afterward, the mixture was washed 3 times with lysis buffer, and RNA was extracted using 1 mL of TRIzol reagent for subsequent analysis. The RNA yield and quality were assessed by NanoDrop spectrophotometer, and RT-qPCR was performed to verify the RNA-protein interaction.

### Sucrose density gradient centrifugation

Cells were treated with 100 μg/mL cycloheximide (Sigma-Aldrich, C7698), washed with PBS, and collected by centrifugation at 101*g* for 5 minutes at 4°C. The cell pellet was resuspended in 1 mL of cold extraction buffer containing 20 mM Tris-HCl (pH 7.5), 150 mM NaCl, 1% NP-40, 1 mM DTT, 1 mM EDTA, protease inhibitors (Sigma-Aldrich, P8340), and RNase inhibitors (Thermo Fisher Scientific, EN0531), followed by incubation on ice for 30 minutes with gentle agitation to lyse the cells. The lysate was centrifuged, and the supernatant was used for gradient preparation. A sucrose gradient consisting of 10%, 20%, 30%, 40%, and 50% concentrations was prepared by layering the solutions in ultracentrifuge tubes and incubating at 4°C overnight. The lysate was carefully layered onto the gradient, and centrifugation was performed at 190,000*g* for 90 minutes at 4°C. After centrifugation, the gradient was fractionated into 18 parts, and RNA was extracted from each fraction using TRIzol reagent according to the manufacturer’s instructions. The RNA was then analyzed by RT-qPCR to quantify specific RNA levels in each fraction. Ct values were calculated, and the distribution of RNA across the gradient was plotted to generate a curve representing the relative abundance of RNA in each fraction.

### Actinomycin D experiment

Cells were seeded in 6-well plates for 24 hours and treated with 5 μg/mL actinomycin D solution (Sigma-Aldrich, A1410). Samples were collected at 0, 2, and 4 hours after treatment to assess RNA stability. For RNA extraction, the culture medium was discarded, and cells were washed with PBS to remove excess serum. One milliliter of TRIzol was added to each well, and the cells were homogenized with RNase-free tips. The mixture was allowed to stand at room temperature for 5 minutes, and the liquid was transferred to RNase-free 1.5 mL EP tubes. The remaining RNA extraction steps followed the RNA extraction procedure described earlier in this section as part of the RT-qPCR analysis. Target RNA levels were assessed by RT-qPCR.

### Statistics

All histograms and line charts in this study were created using GraphPad Prism 9.0, while data analysis was performed with SPSS software version 24.0. The Shapiro-Wilk test was employed to assess normality, and Levene’s test was used to check for homogeneity of variance. For comparisons between 2 unpaired groups, parametric data were analyzed using a 2-tailed Student’s *t* test, and nonparametric data were assessed using the Mann-Whitney test. For comparisons among 3 or 4 groups, 1-way ANOVA was applied, followed by LSD post hoc analysis when *F* ratios were significant (*P* > 0.05) or Dunnett’s test when *F* ratios were not significant (*P* < 0.05). Two-way ANOVA with Tukey’s multiple-comparison test was used to determine significant differences in behavioral parameters. No outlier tests were performed, and no data points were excluded. A *P* value of less than 0.05 was considered statistically significant.

### Study approval

All animal care and experimental procedures were performed according to the NIH *Guide for the Care and Use of Laboratory Animals* (National Academies Press, 2011) and approved by the Ethics Committee of Experimental Animal Center of Xuzhou Medical University (approval no. SYXK2005-0018).

### Data availability

All data supporting the findings of this study are available within the article and its supplemental materials. In addition, all underlying data values for graphs and reported means in both main and Supplemental figures are provided in the Supporting Data Values file, with separate tabs corresponding to each figure panel.

## Author contributions

RY, XL, and MZ designed the research studies. MZ, YL, CG, YL, and LH conducted experiments. MZ and ZF acquired data. XL and XW analyzed data. XL and RY wrote the manuscript. All authors approved the final version of the manuscript.

## Supplementary Material

Supplemental data

Unedited blot and gel images

Supporting data values

## Version 1 08/08/2025

Electronic publication
