# Supplementary material for: NAT10-mediated ac4C modification of Lipin1 mRNA contributes to the pathogenesis of PWMI
Source: JCI Insight. 2025 Aug 8;10(15):e193712. doi: 10.1172/jci.insight.193712 (PMC12333955; doi:10.1172/jci.insight.193712)
Supplement: Supplemental data [file jciinsight-10-193712-s151.pdf]

Supplementary Figure 1

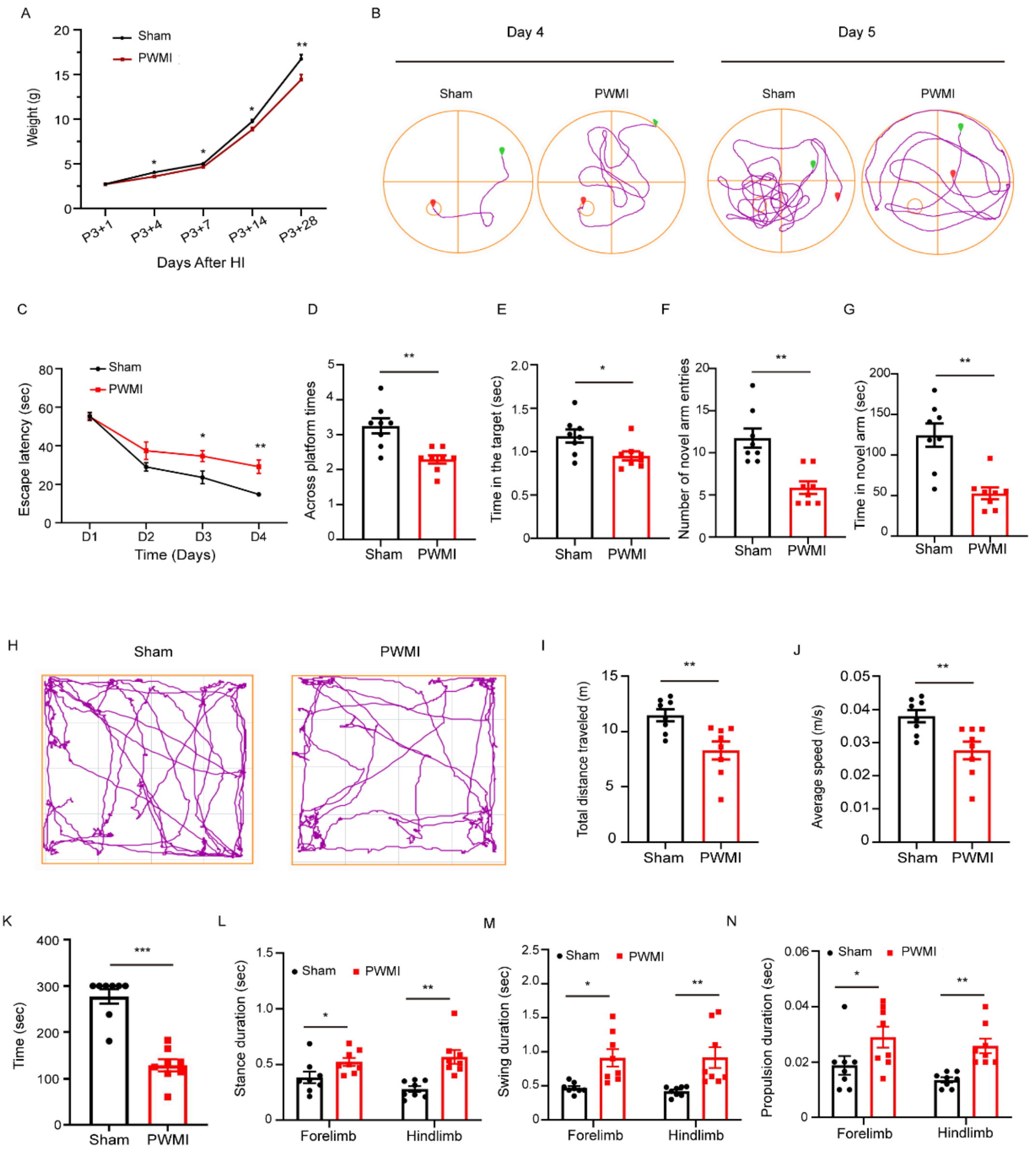

Supplementary Figure 1. Impaired Spatial Memory and Motor Function in PWMI Model Mice. A: Comparison of weight gain between PWMI group and Sham group. B: Representative trajectory plots of both groups of mice on days 4 and 5 of the Morris water maze task. C: The average time spent to locate the hidden platform during the first four days of the spatial navigation training in both groups of mice. D-E: The number of crossings and the time spent in the original platform area during the spatial probe test on day 5 in both groups of mice. F-G: The number of entries into the novel arm and the time spent in the novel arm in the Y-maze test for both groups of mice. H: Representative trajectory plots of both groups of mice in the open field test. I-J: Total distance traveled and average speed of both groups of mice in the open field. K: The time spent on the rotating rod by both groups of mice. L-N: Gait analysis of both groups of mice, including the duration of stance, swing, and propulsion phases for the forelimbs

and hindlimbs. n = 8. Statistics, two-tailed Student's t-test (A, D, E, G, I, J, L, M, N), two-way ANOVA with Tukey's multiple comparisons test (C) and Mann-Whitney test (F, K). The results were expressed as Mean ± SEM, * $P < 0.05$, ** $P < 0.01$, *** $P < 0.001$.

Supplementary Figure 2

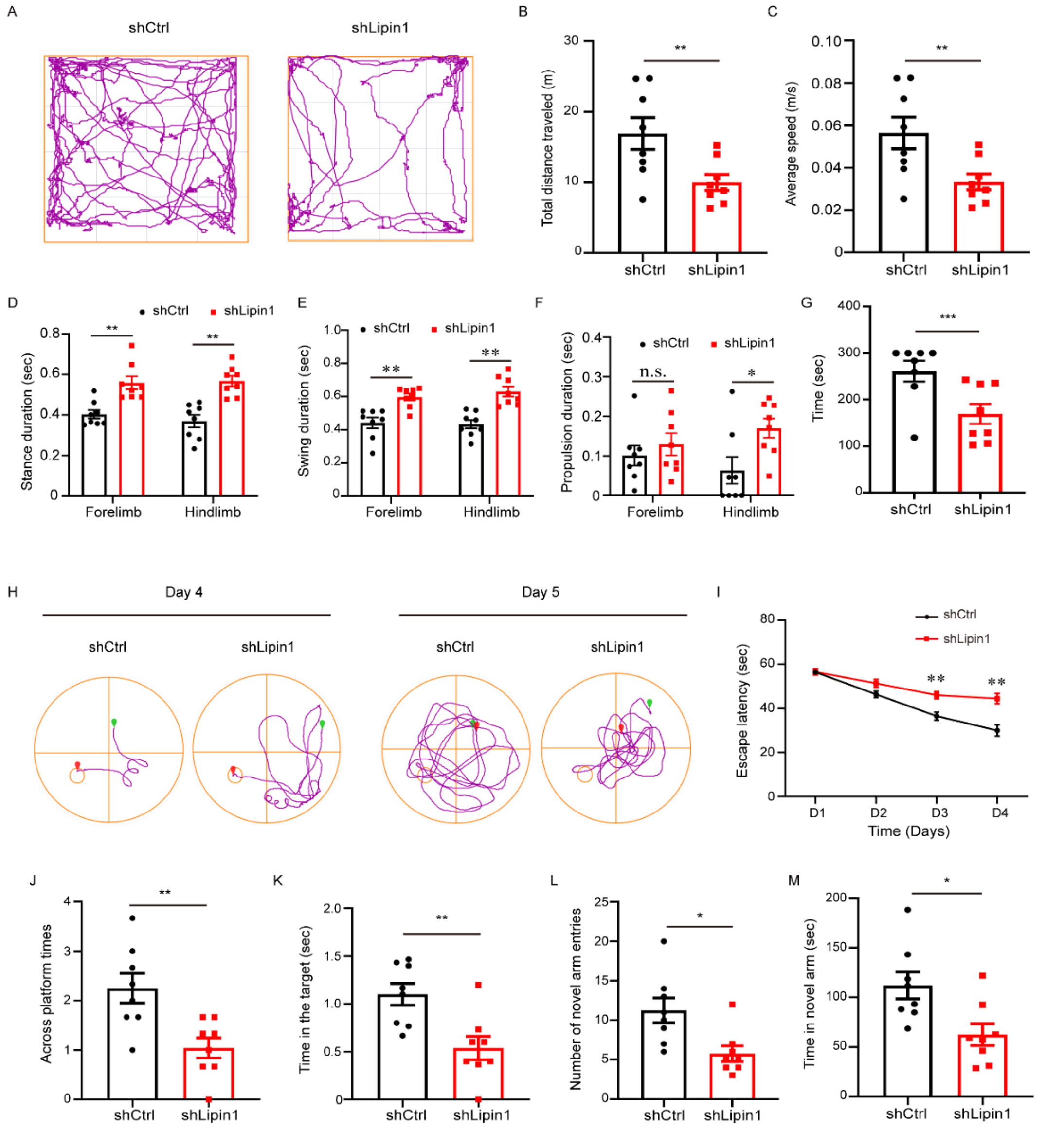

Supplementary Figure 2. Downregulation of Lipin1 in Vivo in the Corpus Callosum Resulted in Impaired Spatial Memory and Motor Function in Mice. A: Representative track diagram showing the movement of mice from both groups in the open field. B-C: Total distance traveled and average speed of mice from both groups in the open field. D-F: Gait analysis showing the standing duration, swing duration, and propulsive duration of the forelimb and hindlimb in both groups of mice. G: Duration of time spent by mice from both groups on the rotating rod. H: Morris water maze experiment on days 4 and 5, showing the representative trajectory of mice from both groups. I: The average time taken by both groups of mice to locate the hidden platforms during the first 4 days of the orientation navigation training. J-K: The number of times both groups of mice crossed the original platform and the time spent on it during the 5th day of the spatial exploration experiment. L-M: In the Y-maze experiment, the number of entries into the new arm and the time spent in the new arm by both groups of mice. Statistics, two-tailed Student's t-test (B, C, F, J, K, M), Mann-Whitney test (D,

E, G, L) and two-way ANOVA with Tukey's multiple comparisons test (I). The results were expressed as Mean ± SEM, n = 8, * $P < 0.05$, ** $P < 0.01$, *** $P < 0.001$.

Supplementary Figure 3

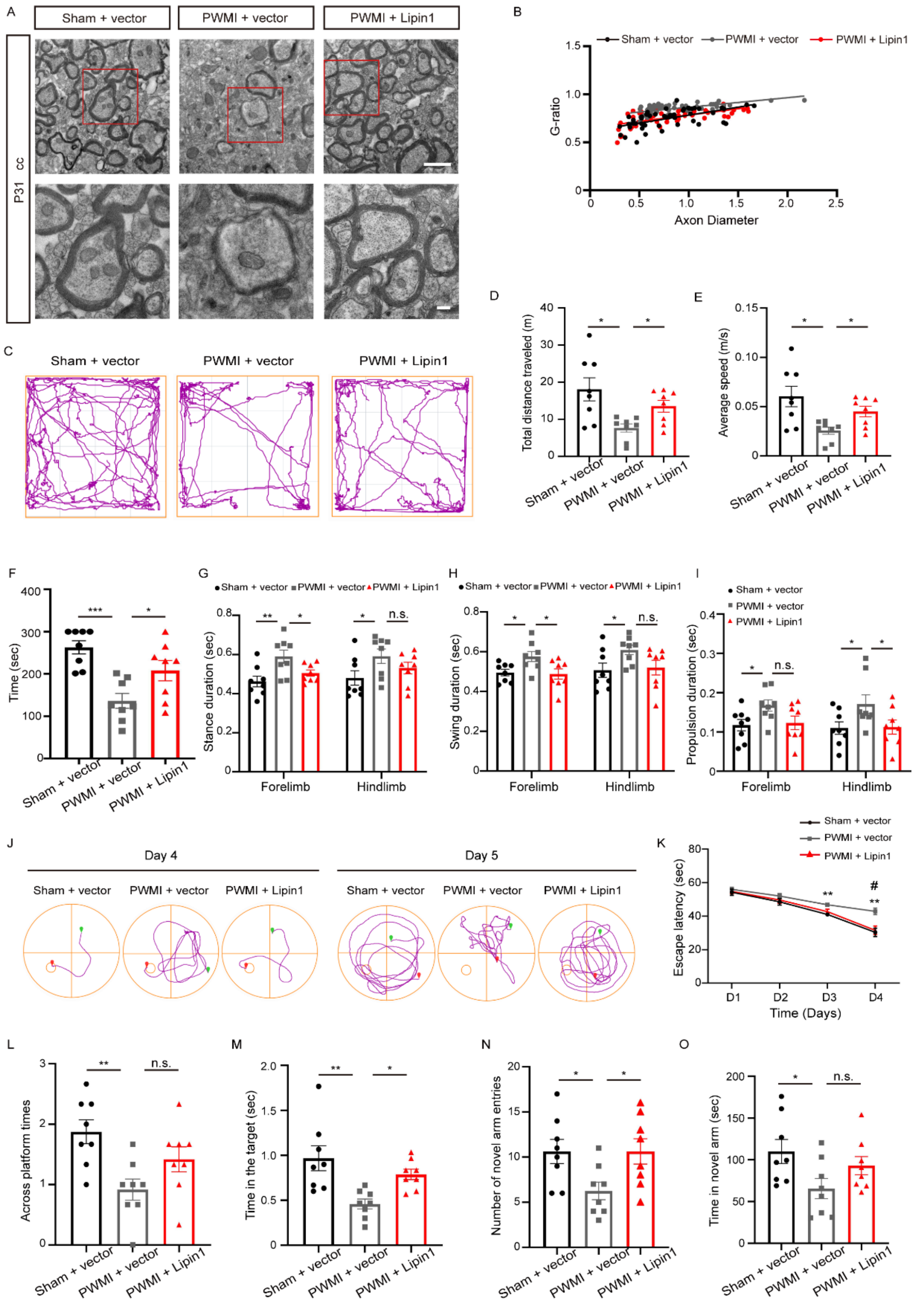

Supplementary Figure 3. Overexpression of Lipin1 in the Corpus Callosum Improves Spatial Memory and Motor Function in PWMI Mice. A: TEM images of myelin in the corpus callosum, with high magnification images shown as insets of low magnification images. Low magnification Bar = 1 μm, high magnification Bar = 200 nm. B: Distribution and statistical analysis of G-ratio of myelinated axons in the three

groups of mice. n = 3. C-E: Representative track diagram, total distance, and average speed of the three mouse groups in the open field. F: The time spent by the three mouse groups on the rotating rod. G-I: Gait analysis measured the standing, swing, and propulsive durations of the forelimb and hindlimb in the three mouse groups. J: Morris water maze experiment on days 4 and 5, showing the representative trajectory of the three mouse groups. K: The average time taken by the three mouse groups to locate the hidden platform during the first 4 days of orientation navigation training, * Sham + vector vs. PWMI + vector; # PWMI + vector vs. PWMI + Lipin1. L-M: The number of times the three mouse groups crossed the original platform and the time spent on it during the space exploration experiment on day 5. N-O: In the Y-maze experiment, the number of entries into the new arm and the time spent in the new arm by the three mouse groups. n = 8. Statistics, one-way ANOVA with LSD post-hoc analysis (D, E, F, G, H, I, L, M, N, O) and two-way ANOVA with Tukey's multiple comparisons test (K). The results were expressed as Mean ± SEM, * $P < 0.05$, ** $P < 0.01$, *** $P < 0.001$.

Supplementary Figure 4

A

Primer pair 1

	Sequence (5'->3')	Length	Tm	GC%	Self complementarity	Self 3' complementarity
**Forward primer**	AAGAGACTGACAACGATCAGGA	22	58.84	45.45	4.00	2.00
**Reverse primer**	TTCCCCAGAGAACCAGTGGAT	21	60.49	52.38	5.00	5.00

Products on target templates

>NM_001130412.1 Mus musculus lipin 1 (Lpin1), transcript variant 3, mRNA

```
product length = 190
Forward primer  1    AAGAGACTGACAACGATCAGGA  22
Template        475  ......................  496

Reverse primer  1    TTCCCCAGAGAACCAGTGGAT  21
Template        664  .....................  644
```

B

Primer pair 1

	Sequence (5'->3')	Length	Tm	GC%	Self complementarity	Self 3' complementarity
**Forward primer**	GGAGAGTGTTTCCTCGTCCC	20	59.75	60.00	4.00	1.00
**Reverse primer**	ATGAAGGGGTCGTTGATGGC	20	60.39	55.00	3.00	2.00

Products on target templates

>NM_001289726.2 Mus musculus glyceraldehyde-3-phosphate dehydrogenase (Gapdh), transcript variant 1, mRNA

```
product length = 136
Forward primer  1    GGAGAGTGTTTCCTCGTCCC  20
Template        84   ....................  103

Reverse primer  1    ATGAAGGGGTCGTTGATGGC  20
Template        219  ....................  200
```

Supplementary Figure 4. Verification of primer specificity by NCBI Primer-BLAST. A-B: Representative screenshots of Primer-BLAST results for the *Lpin1* and *Gapdh* primers used in RT-qPCR. The analyses confirm that each primer pair generates a single, specific amplicon corresponding to the target gene in *Mus musculus*, without significant off-target alignments. Target transcript IDs include *Lipin1* (GenBank: NM_001130412.1) and *Gapdh* (GenBank: NM_001289726.2).

Supplementary Table S1 List of Lipin1 mRNA-associated Proteins Identified by Mass Spectrometry

RNA pull down	Peptide (No.)
RNA helicase (Ddx3x)	13
Serpine1 mRNA binding protein 1 (Serbp1)	8
Small ribosomal subunit protein (RACK1)	7
RRM domain-containing protein (Hnrnpc)	6
RNA cytidine acetyltransferase (Nat10)	4
DNA-(apurinic or apyrimidinic site) endonuclease (Apex1)	4
Probable ATP-dependent RNA helicase (DDX6)	4
CCN family member 1 (Ccn1)	4
Keratin, type I cytoskeletal 13 (Krt13)	4

Supplementary Table S2 Primer Sequences and qPCR Parameters

Gene	Forward Primer (5' to 3')	Reverse Primer (5' to 3')	Amplicon Size (bp)	GenBank Accession
Lipin1	AAGAGACTGACAACGATCAGGA	TTCCCCAGAGAACCAGTGGAT	190	NM_001130412.1
Gapdh	GGAGAGTGTTTCCTCGTCCC	ATGAAGGGGTCGTTGATGGC	136	NM_001289726.2

Supplementary Table S3 List of Lipin1 shRNA and its blank vector sequences

Name	Sequence 5’ to 3’
Lipin1 shRNA	GGAGACAACGGAGAAGCAT
Control shRNA	GTAGCGCGGTGTATTATAC
