# Supplementary material for: NAT10-mediated ac4C modification of Lipin1 mRNA contributes to the pathogenesis of PWMI
Source: JCI Insight. 2025 Aug 8;10(15):e193712. doi: 10.1172/jci.insight.193712 (PMC12333955; doi:10.1172/jci.insight.193712)

Figure 1D

Sham MBP

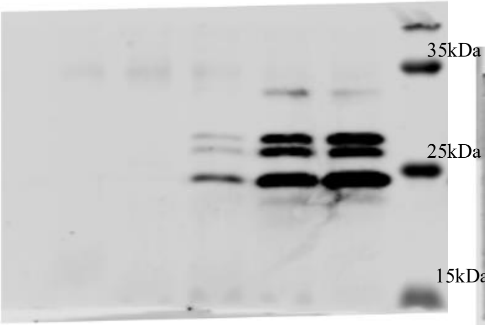

Sham β-actin

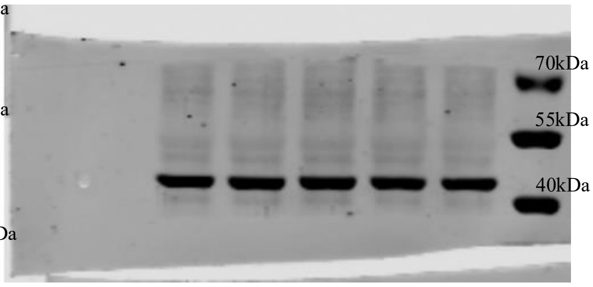

HI (L) MBP

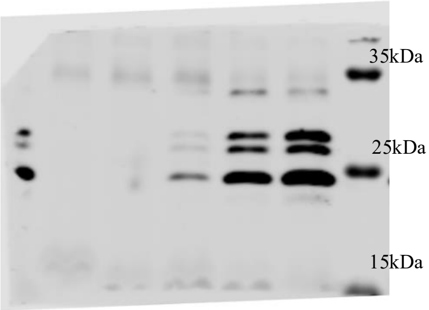

HI (L) β-actin

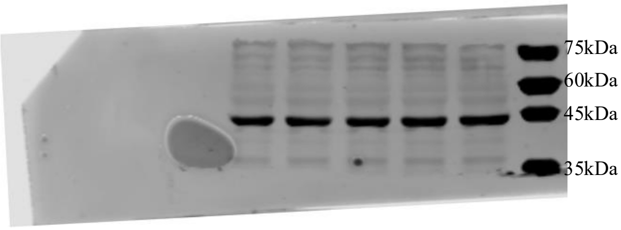

HI (R) MBP

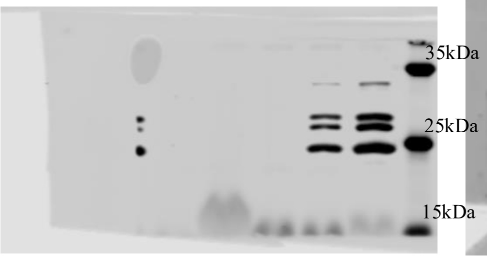

HI (R) β-actin

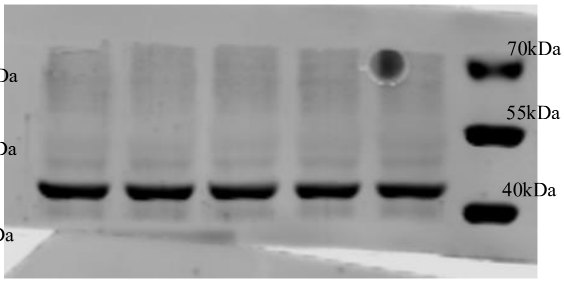

Figure 1L

Sham PDGFR-α

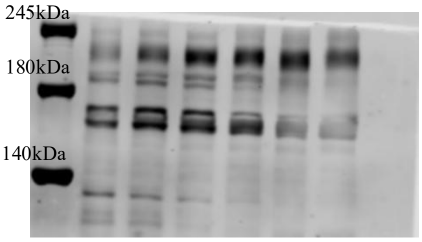

Sham β-actin

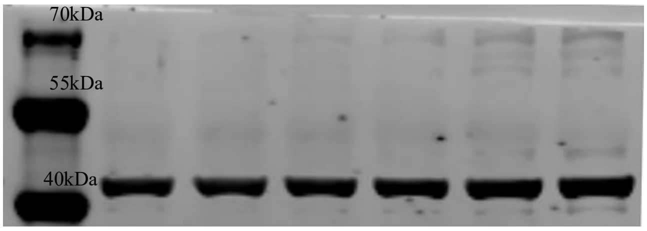

HI (L) PDGFR-α

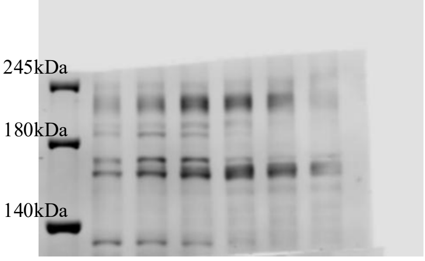

HI (L) β-actin

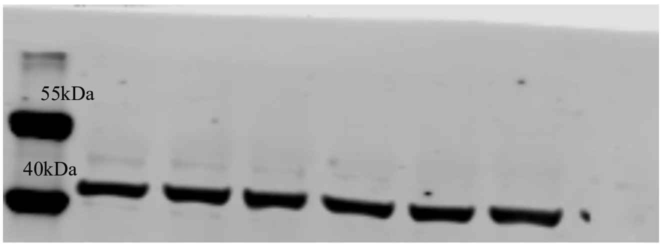

HI (R) PDGFR-α

HI (R) β-actin

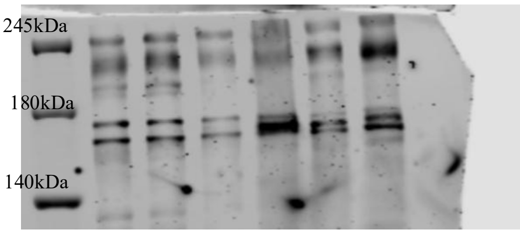

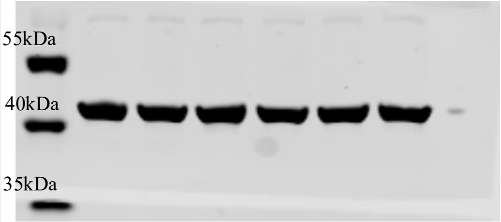

Figure2B

PWMI Lipin1

Sham Lipin1

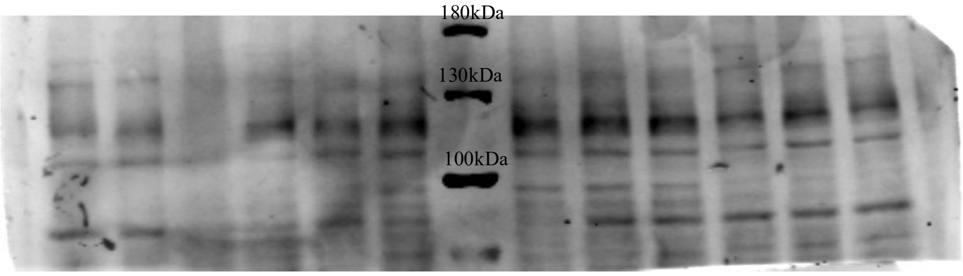

Sham NC

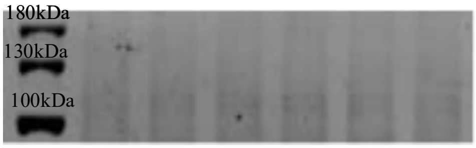

PWMI NC

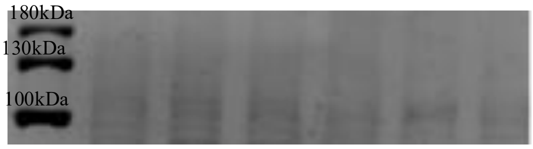

PWMI β-actin

Sham β-actin

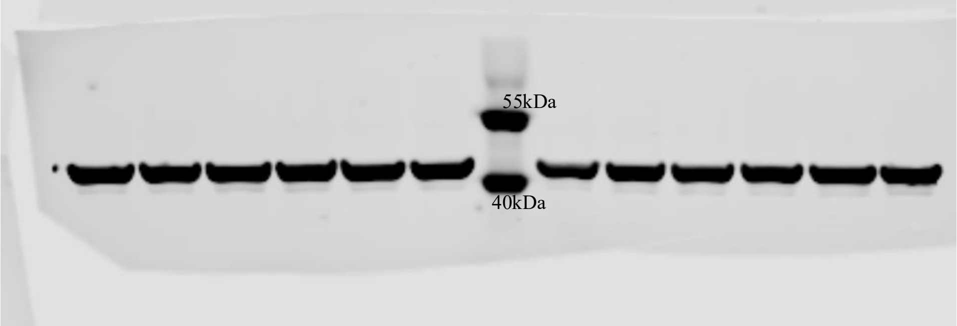

Figure 2I

Lipin1

β-tubulin

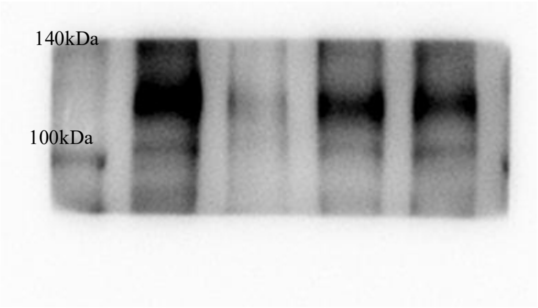

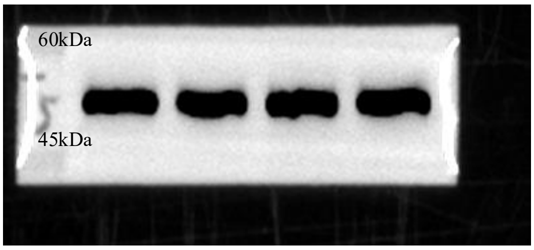

Figure 3C

Lipin1

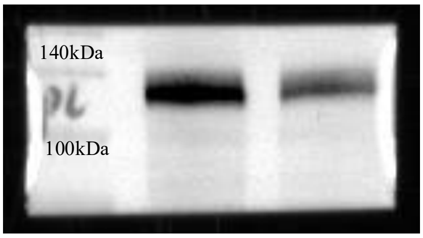

β-tubulin

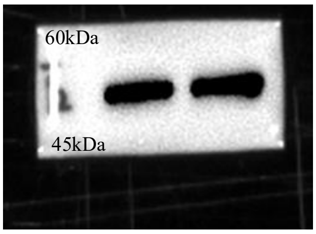

MBP

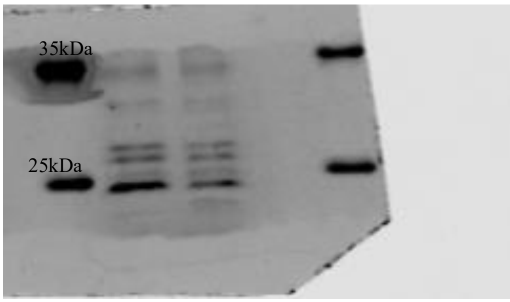

β-actin

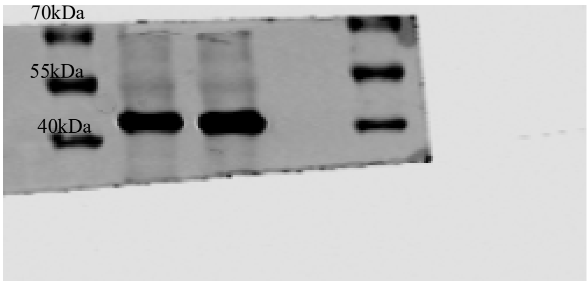

CC1

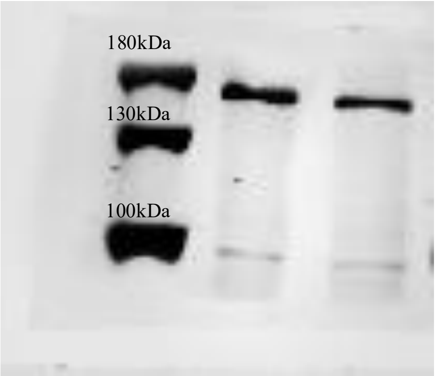

β-actin

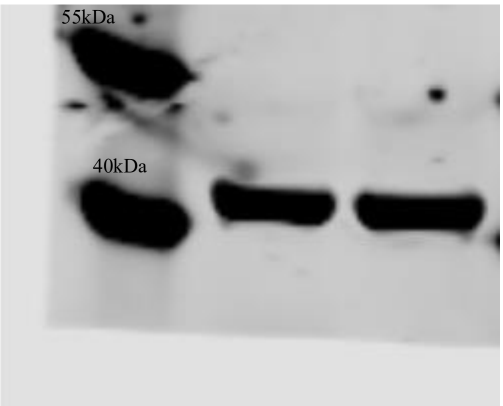

PDGFR-α

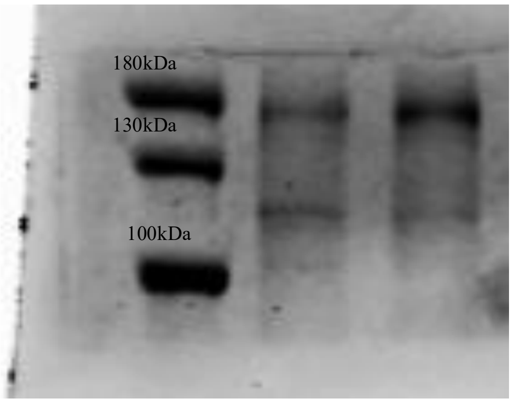

β-actin

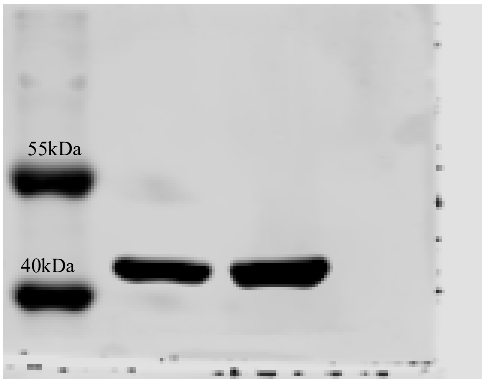

Figure 4B

Lipin1

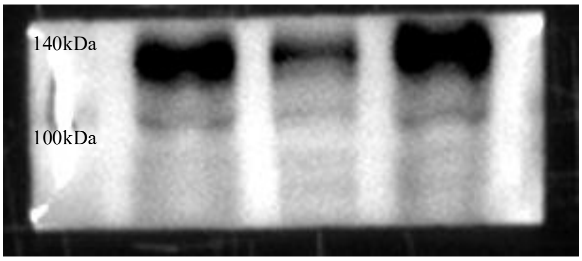

β-tubulin

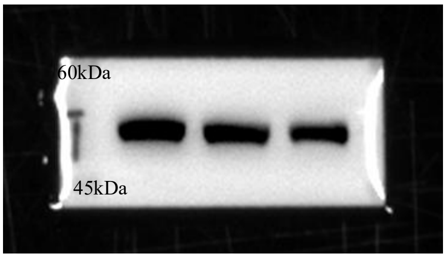

CC1

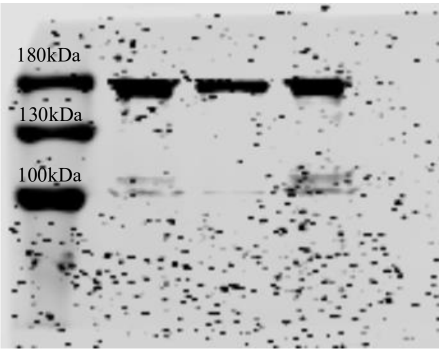

β-actin

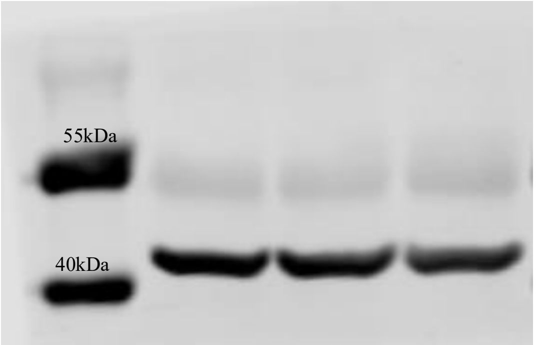

PDGFR-α

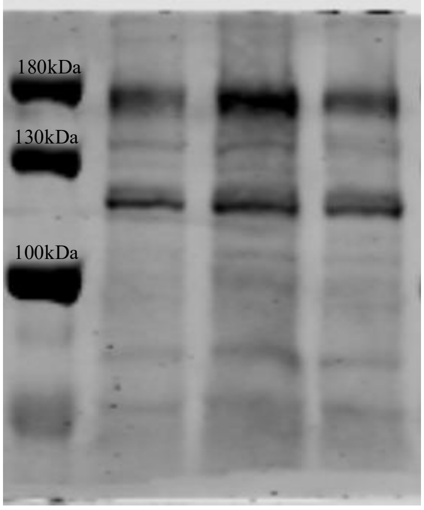

β-actin

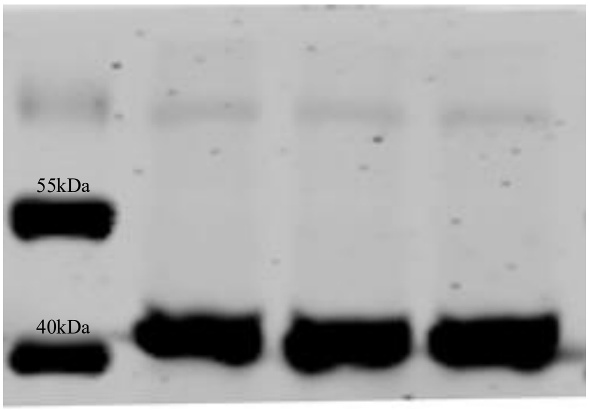

Figure 5G

NAT10

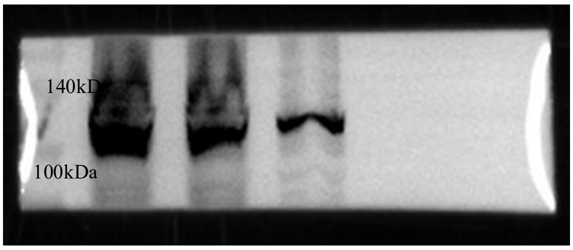

β-tubulin

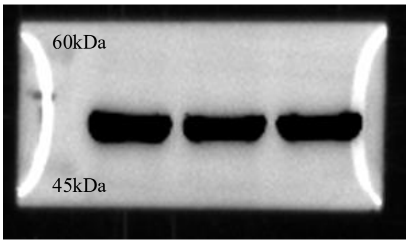

NC

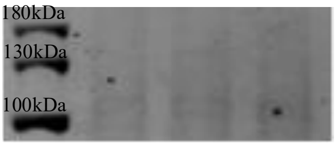

Figure 5K

NAT10

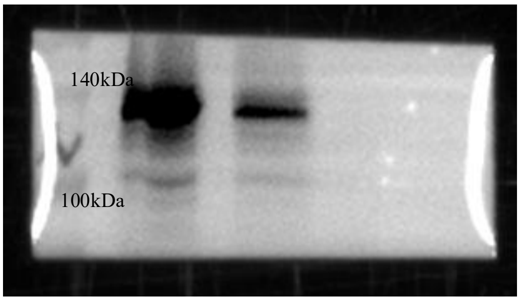

β-tubulin

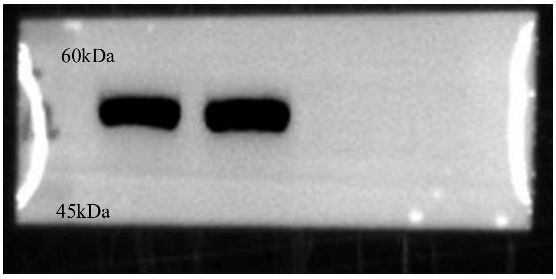

Figure 6A

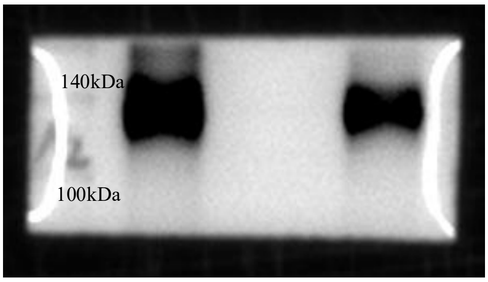

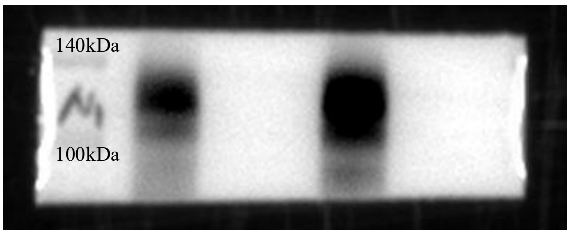

Figure 6B

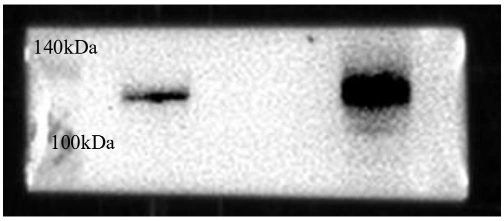

Figure 6D

NAT10

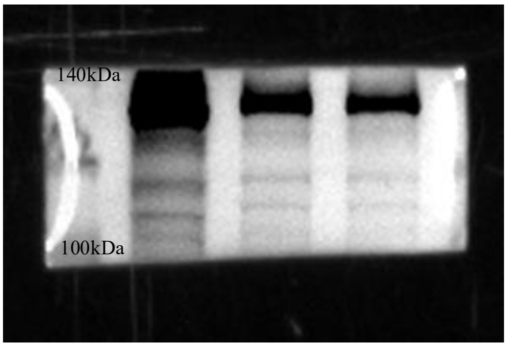

β-tubulin

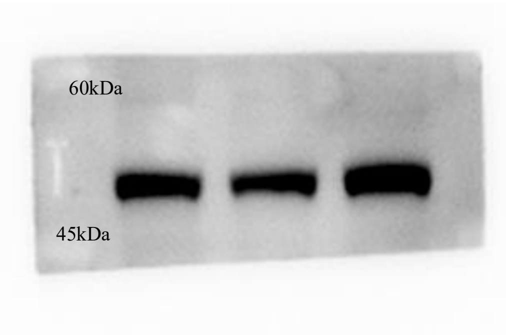

Lipin1

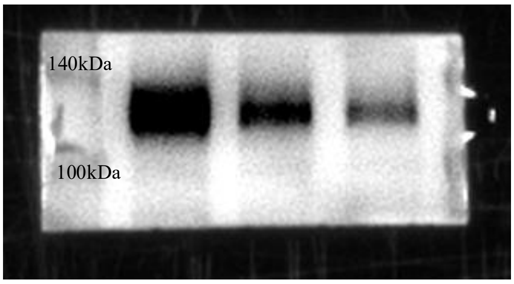

β-tubulin

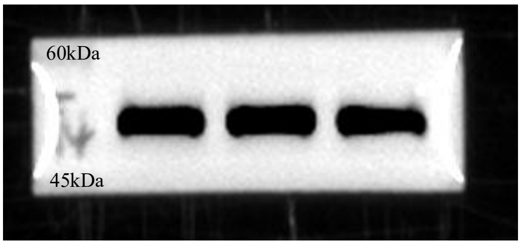

Figure 7E

MBP

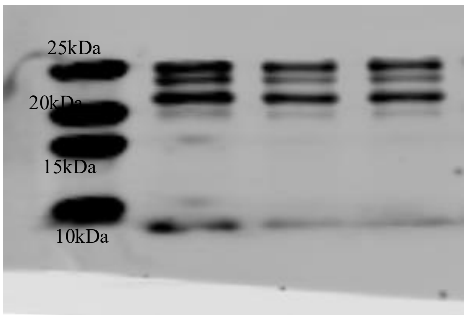

β-actin

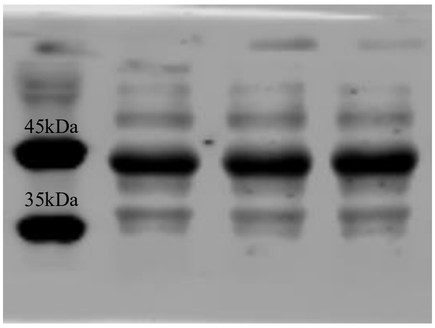

PDGFR-α

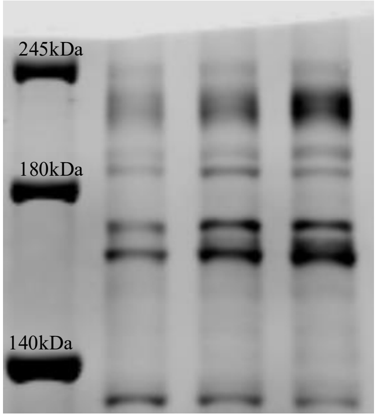

β-actin

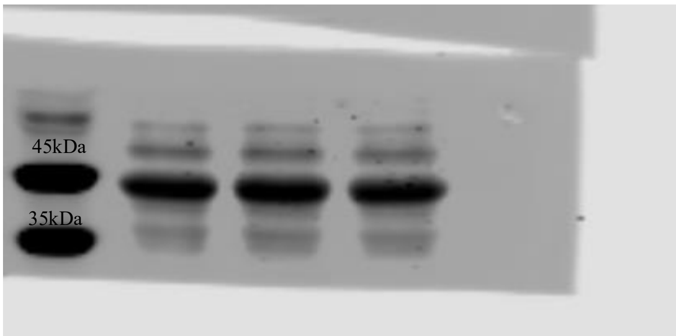

Figure 7G

NAT10

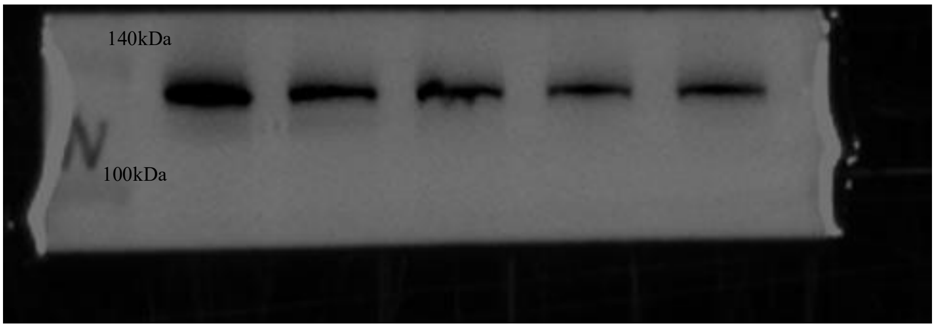

Lipin1

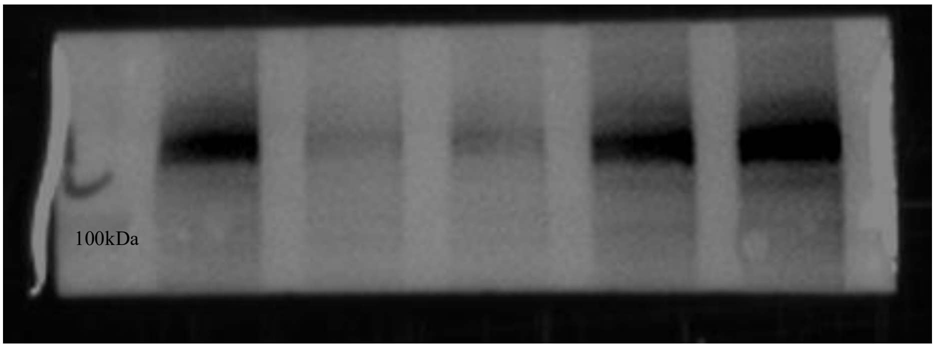

PDGFR-α

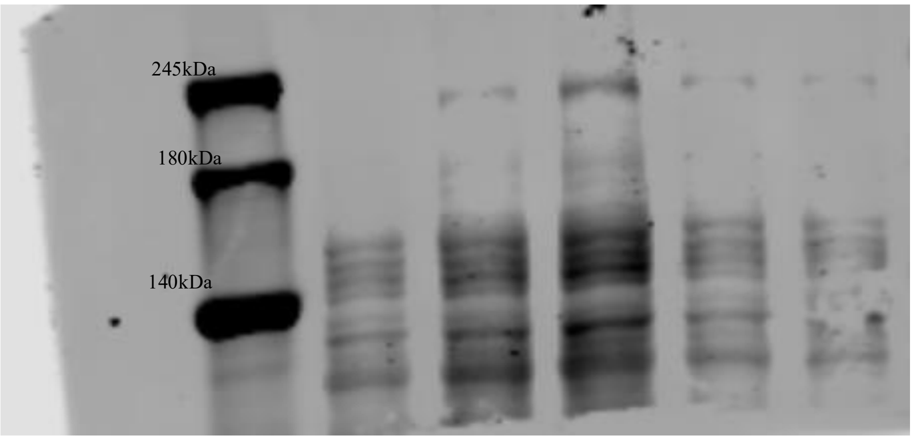

MBP

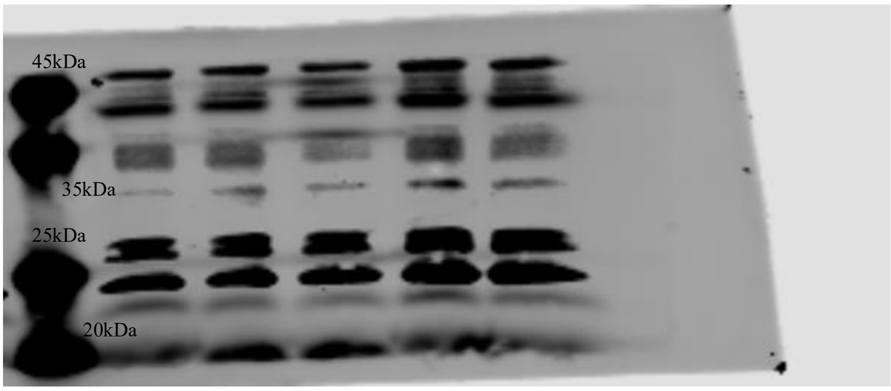

β-tubulin

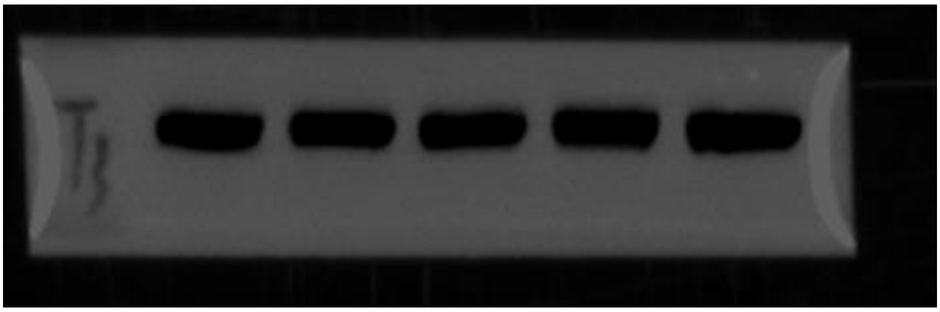

Supplement: Unedited blot and gel images [file jciinsight-10-193712-s152.pdf]
